# Design of a Prospective Human–Animal Cohort Study to Evaluate the Role of Camels and Other Livestock Species in the Transmission of *Brucella* spp. to Humans in Kenya

**DOI:** 10.3390/ijerph22121859

**Published:** 2025-12-12

**Authors:** Dismas Oketch, Ruth Njoroge, Isaac Ngere, John Gachohi, Samuel Waiguru, Dalmas Omia, Peninah Munyua, Samoel Khamadi, Bonventure Juma, Athman Mwatondo, Samson Limbaso, Mathew Muturi, Roland Ashford, Adrian Whatmore, John McGiven, Scott Nuismer, Felix Lankester, John Njeru, Ali Boru, Boku Bodha, Lydia Kilowua, Nazaria Nyaga, Humphrey Njaanake, Walter Jaoko, Kariuki Njenga, Eric Osoro

**Affiliations:** 1 Department of Medical Microbiology and Immunology, University of Nairobi, Nairobi 00200, Kenya; kn@uonbi.ac.ke (H.N.); wjaoko07@gmail.com (W.J.); 2 Washington State University Global Health Program, Washington State University, P.O. Box 72938, Nairobi 00200, Kenya; ruth.njoroge@wsu.edu (R.N.); isaac.ngere@wsu.edu (I.N.); john.gachohi@wsu.edu (J.G.); waigurusamuel@uidaho.edu (S.W.); mkariuki.njenga@wsu.edu (K.N.); eric.osoro@wsu.edu (E.O.); 3 Paul G. Allen School for Global Health, Washington State University, Pullman, WA 99163, USA; felix.lankester@wsu.edu; 4 School of Public Health, Jomo Kenyatta University of Agriculture and Technology, Nairobi 00200, Kenya; 5 Department of Biological Sciences, College of Science, University of Idaho, Moscow, ID 83844, USA; snuismer@uidaho.edu; 6 Department of Anthropology, Gender and African Studies, University of Nairobi, Nairobi 00200, Kenya; ochiengomia@uonbi.ac.ke; 7 Division of Global Health Protection, Centers for Disease Control and Prevention, Nairobi 00621, Kenya; munyuap@gmail.com (P.M.); xwl2@cdc.gov (B.J.); 8 Centre for Virus Research, Kenya Medical Research Institute, Nairobi 00200, Kenya; skhamadi@kemri.go.ke (S.K.); limbaso@gmail.com (S.L.); 9 Zoonotic Disease Unit, Ministry of Health, Nairobi 00200, Kenya; amwatondo@gmail.com (A.M.); muturimathew@gmail.com (M.M.); 10 Department of Veterinary Medicine, Dahlem Research School of Biomedical Sciences (DRS), Freie Universität Berlin, 14195 Berlin, Germany; 11 Department of Bacteriology, Animal and Plant Health Agency (APHA), Weybridge KT15 3NB, UK; roland.ashford@apha.gov.uk (R.A.); adrian.whatmore@apha.gov.uk (A.W.); 12 Department for International Development, Innovation and Business, Animal and Plant Health Agency (APHA), Weybridge KT15 3NB, UK; john.mcgiven@apha.gov.uk; 13 Global Health Tanzania, Arusha P.O. Box 1640, Tanzania; 14 Centre for Microbiology Research, Kenya Medical Research Institute, Nairobi 00200, Kenya; mwanikij@gmail.com; 15 Department of Health Services, County Government of Marsabit, Marsabit 60500, Kenya; aliboru27@gmail.com; 16 Department of Veterinary Services, County Government of Marsabit, Marsabit 60500, Kenya; bokubodha@gmail.com; 17 Department of Health, County Government of Kajiado, Kajiado 01100, Kenya; lmunteyian@gmail.com; 18 Department of Veterinary Services, County Government of Kajiado, Kajiado 01100, Kenya; wanjanyaga67@gmail.com; 19 KAVI Institute of Clinical Research, University of Nairobi, Nairobi 00200, Kenya

**Keywords:** *Brucella*, brucellosis, zoonosis, epidemiology, study protocol, transmission, livestock, one health

## Abstract

Brucellosis remains a major zoonotic disease worldwide, with disproportionate burden in low- and middle-income countries where limited veterinary and healthcare infrastructure constrain effective control measures. However, its pathways of transmission are poorly understood. In pastoralist settings, we hypothesize that camels have a high burden of *Brucella* spp. and play a key role in spreading it to humans and other livestock. This manuscript presents a study protocol to quantify the relative contribution of various livestock species to brucellosis transmission and identify cost-effective control strategies in Kenya. Using probability-proportional-to-size sampling, we aimed to recruit a longitudinal cohort of 170 households and their herds per site in the Marsabit and Kajiado counties. Households rearing at least one livestock species (cattle, camels, goats, sheep) were eligible. Serum, milk, and vaginal swabs (from livestock), and serum (from humans) were collected for testing using Rose Bengal Test, ELISA, qPCR, and culture methods. Concurrently, surveillance for suspected brucellosis was conducted in study health facilities. A qualitative ethnographic study and livestock movement monitoring using GPS-collared animals were nested within the cohort. These data will be used to parameterize a multi-host, multi-species infectious disease model through Approximate Bayesian Computation. Through this One Health approach, our study will identify and optimize potential interventions and help inform the development of a comprehensive cost-effective national control program for brucellosis.

## 1. Introduction

Brucellosis is the most common zoonotic disease worldwide, with more than 2.1 million new human cases reported annually [1]. While brucellosis has been controlled in humans in the USA, Europe, and Australia through effective livestock vaccination and culling programs, it remains endemic in many low- and middle-income countries in Africa, Latin America, Central Asia, and the eastern Mediterranean [2,3,4,5]. Within these endemic areas, brucellosis causes serious public health impacts and results in enormous economic losses, due to reduced livestock productivity, and a significant public health burden [6,7,8].

*Brucella* spp. are predominantly transmitted to livestock via direct contact with abortion products, birth fluids and tissues from infected animals [9,10]. Human infection occurs primarily through consumption of unpasteurized milk and dairy products, or direct contact with infected animal tissues, birth fluids, and handling of sick animals, representing distinct but overlapping transmission pathways between livestock and humans [11,12]. The characteristic clinical signs of brucellosis in livestock include mid- to late-gestation abortions, stillbirths, infertility and delivery of weak offspring [11]. Offspring of infected dams can become persistently infected and remain seronegative until they abort, at which time they seroconvert and may shed brucellae into the environment [3]. The signs and symptoms of human brucellosis are nonspecific and highly variable, and include undulating fever, malaise, fatigue, anorexia, sweating, and joint pains, inter alia.

Without appropriate antibiotic treatment, progression to chronic brucellosis occurs in 11–30% of acute cases, with variability influenced by timely diagnosis, treatment regimen optimization, and patient-specific risk factors, including joint pain and renal function impairment [13,14]. Following treatment, 5–15% of treated cases relapse [15,16]. Recent therapeutic advances, particularly triple antibiotic regimens, have substantially reduced chronicity, relapse and therapeutic failure rates compared to historical data [17].

There are currently twelve recognized Brucella species: *B. melitensis*, *B. abortus*, *B. suis*, *B. canis*, *B. ovis*, *B. neotomae*, *B. ceti*, *B. pinnipedialis*, *B. microti*, *B. inopinata*, *B. papionis* and *B. vulpis* [18,19]. *B. abortus* and *B. melitensis* are the predominant species associated with human disease, with more than 70% of cases worldwide caused by *B. melitensis* [5,11]. Although livestock are the primary source of human infection, wild animals may act as reservoirs in regions with frequent human–wildlife interactions [20,21,22], with the possibility of livestock serving as a “bridge population” that can potentially transmit the pathogens between wildlife and humans.

Zoonotic pathogens such as *Brucella* spp. are highly prevalent among humans and livestock in the arid and semi-arid lands (ASALs) in northern Kenya and the Horn of Africa [23,24,25,26,27,28,29], with strong animal–human association [28,30] and significant socioeconomic impact [8]. According to the “National Strategy for the Prevention and Control of Brucellosis in Humans and Animals in Kenya (2021–2040)”, seroprevalence studies indicate that human brucellosis rates range from 0.6% to 46%, depending on geographical location, population risk factors, and diagnostic methods used [31]. Pastoralist communities, particularly in counties such as Marsabit and Kajiado, are disproportionately affected due to their close interaction with livestock and traditional practices such as raw milk consumption. National surveys and localized studies report animal-level seroprevalences of 2% to 35% in cattle, goats, and camels, with higher rates in extensive, communal grazing systems compared to intensive, commercial farming operations [32].

In a human–animal linked household study conducted in the Kiambu, Kajiado, and Marsabit counties in Kenya in 2013–2014, the household and herd brucellosis seroprevalence ranged from 5% to 73% and 6% to 68%, respectively, with highest seroprevalence reported in Marsabit [28]. When the study compared seroprevalence between two ASAL counties, Marsabit and Kajiado, it found a 2.5-fold higher household/herd prevalence and 4-fold higher individual livestock and human prevalence in Marsabit. Most households in Marsabit had camels, whereas those in Kajiado did not. Equally, Akoko et al. [23] reported high *Brucella* spp. prevalence in livestock (33.3%) and humans (38.5%) in a cross-sectional study to assess host–pathogen associations in multi-host livestock populations in the Narok and Marsabit counties in Kenya. They also detected both *B. abortus* and *B. melitensis* DNA in humans and in multiple livestock host species—suggesting cross-species transmission. *B. abortus* was the predominant species affecting cattle and camels while *B. melitensis* was predominant in small ruminants.

Since its first description in camels in 1931, brucellosis remains one of the most widespread infections among camels worldwide [29,33]. Traditionally, camels are not considered primary hosts of *Brucella* spp. [10,34], but are known to be susceptible to *B. abortus* and *B. melitensis*, though often with few clinical signs. The epidemiology of brucellosis in camels is poorly understood [34]. Among pastoral communities, raw camel milk and urine are consumed for their perceived medicinal properties and may compound the risk of *Brucella* spp. transmission to humans [35,36]. Additionally, camels have a longer median life expectancy of about 18 years, which would span several generations of small livestock such as goats and sheep. Hence, an infected camel may maintain the infection in a herd by transmitting *Brucella* spp. to generations of sheep, goats, or cattle in its lifetime [34].

In response to prolonged droughts and related climate-variable conditions in the Horn of Africa, most pastoralists have resorted to camel rearing due to the species’ hardiness and longevity [35,36]. The increase in the camel population has resulted in increased risk and potential transmission of several camel-borne zoonotic pathogens, such as *Brucella* spp., and their potential spillover to humans and other susceptible livestock [29,36].

Recognizing the high burden and enormous impact of brucellosis, Kenya has adopted multiple strategies to control the disease in both livestock and humans through the National Strategy 2021–2040 [31]. The strategy provides a comprehensive, multisectoral framework for controlling and ultimately eliminating brucellosis through integrated disease surveillance and response, strengthening biosecurity prevention practices, mass livestock vaccination, creating public awareness, enhancing diagnostic capabilities at regional and county levels, early/accurate diagnosis and standardized treatment and leveraging research initiatives.

**Figure 1:**
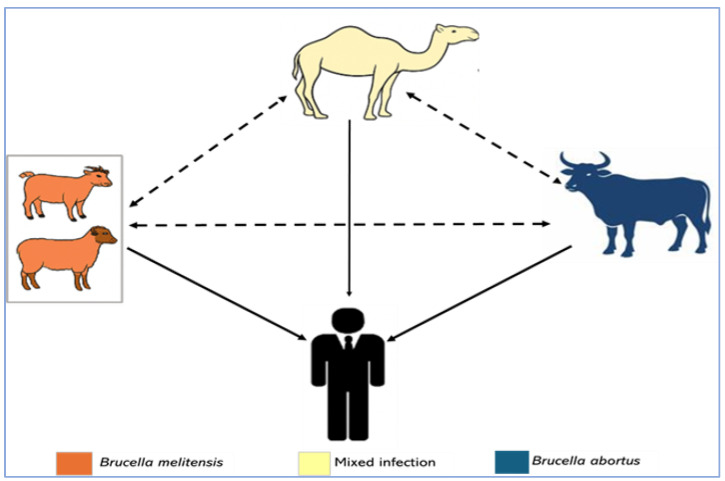
Hypothesized model for transmission of *Brucella* spp. among livestock and to humans. Arrow thickness reflects likely magnitude of transmission. Broken arrows indicate animal–animal transmission and solid arrows indicate animal–human transmission.

Controlling brucellosis in Kenya and sub-Saharan Africa, in general, is constrained in part by the limited understanding of two key epidemiological scenarios: the livestock species that primarily acts as the source for human infections and the *Brucella* species maintained by different livestock hosts. This determination is important because the vaccination of animals is currently the most effective method to control brucellosis, and vaccines are pathogen- and species-specific [37]. Our hypothesis is that camels have a high burden of *Brucella* spp. and may serve as the primary source of infection to humans and other livestock (cattle, sheep, goats) in ASALs regions, as shown in Figure 1. In ASAL counties such as Marsabit and Isiolo, >75% of households rear camels [24], which confirms their regional relevance in the ASALs compared to the non-ASAL regions, such as Kajiado County.

Our study seeks to determine the relative contribution of camels and other livestock (cattle, goats and sheep) to the transmission of brucellosis to humans through a multisite, multidisciplinary approach; and to provide valuable insights into promising intervention strategies for brucellosis control in Kenya. The specific objectives of the study are to (i) estimate the incidence and factors associated with brucellosis in humans and livestock, (ii) quantify and describe the human–animal, inter-herd, and intra-herd livestock interactions that promote transmission of *Brucella* spp., (iii) identify the dominant *Brucella* sp. infecting each livestock species and humans and their pathways of cross-species transmission using molecular typing methodologies, and (iv) identify and optimize effective interventions for brucellosis control and determine the contribution of camels and other livestock in the maintenance and transmission of brucellosis. The study was activated in November 2022. Longitudinal follow-up has been finalized, and study is currently conducting laboratory analyses, data cleaning, and analysis. This manuscript presents the study protocol.

## 2. Materials and Methods

### 2.1. Study Design

**Figure 2:**
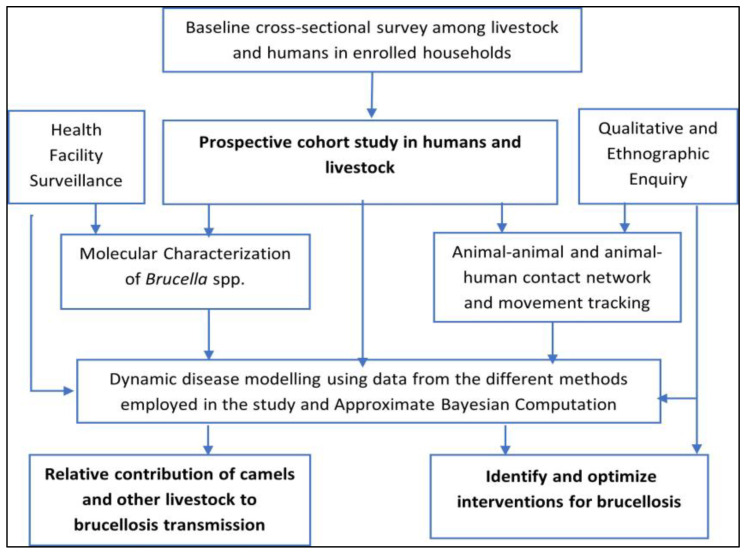
Brucellosis transmission dynamics study schema. Sequential implementation of study objectives leading to a determination of the relative contribution of each livestock species to brucellosis transmission and identification of optimal interventions.

The study employed a three-pronged mixed-methods approach, combining quantitative and qualitative elements, as depicted in Figure 2—Sequential implementation of study objectives leading to a determination of the relative contribution of each livestock to brucellosis transmission and identification of optimal interventions. Central to this was a longitudinal household cohort design targeting humans and livestock within the catchment area of the study health facilities to assess brucellosis incidence and cover the first prong. Concurrently, prospective surveillance for acute febrile illness was conducted in the selected health facilities, focusing on brucellosis testing and case management. The second prong integrated Global Positioning System (GPS) tracking for animal movement assessment and an ethnographic component for qualitative insights into community practices. Using data from this study, we will apply epidemiological modeling through Approximate Bayesian Computation to assess the effectiveness of brucellosis control measures as the third prong. This multi-method and transdisciplinary approach can provide insights often overlooked by singular quantitative or qualitative inquiries on their own.

### 2.2. Study Site and Participants

**Figure 3:**
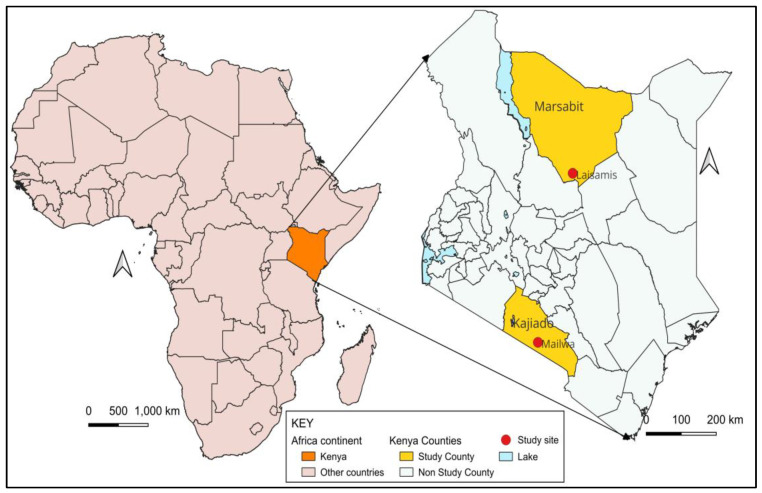
Map of Kenya showing the Marsabit and Kajiado counties and the study sites. Gray—study counties; white—non-study counties; right panel—study reference health facilities. [Map Courtesy: Stella Mamuti].

The study was conducted in two distinct study sites in pastoralist counties in Kenya—Marsabit and Kajiado—chosen for their different demographic and ecological characteristics relevant to brucellosis transmission and camel-rearing status. Both are primarily arid or semi-arid, sparsely populated with similar cultural and pastoralist practices. More than 75% of households in both study sites depend on pastoralism for their livelihoods, providing a critical context for understanding zoonotic disease transmission in arid environments. Cattle, goats, and sheep are reared in both counties, while camels are only reared in Marsabit County, as shown in Supplementary Table S1—Study Site Characteristics. Laisamis Subcounty Referral Hospital and the Mailwa Health Center were selected as the study reference health facilities in the Marsabit and Kajiado counties, respectively (Figure 3). The study reference health facilities required basic infrastructure to support field laboratory diagnostics and initial sample preparation, while the catchment population needed to be pastoralists living in proximity to their livestock.

### 2.3. Study Population and Eligibility Criteria

Our study includes humans and livestock in the community cohort and patients attending selected study health facilities. Households were eligible if they were located within 30 km of the study health facility, reared at least one livestock species (cattle, camels, goats, sheep) and the household head provided informed consent for their household and herd to participate. Once enrolled, households and herds were followed up even if they had moved out of the study area into distant *fora*. Livestock in enrolled households were eligible for inclusion. Human participants were eligible if they were residents of an eligible household for at least the preceding four months, were aged 12 months and above and provided individual informed consent, assent and/or parental permission to participate. Children under 12 months were excluded because of the difficulty in collecting blood samples in community settings. In the health facility surveillance, a participant must have met the suspected brucellosis case definition (see Study Definitions), not be severely ill as determined by the study clinician and lack documented evidence of bleeding disorder.

### 2.4. Study Definitions

*Acute febrile illness (AFI)*: Reported history of hotness of body or measured fever (axillary temperature >38 °C) in the preceding 21 days.

*Suspected brucellosis (Human)*: A case of AFI that is epidemiologically linked to suspected or confirmed animal case AND at least ONE of the following symptoms: night sweats, joint pains/swelling, headache, fatigue, or anorexia.

*Suspected brucellosis (Animal)*: Observed clinical signs of any of the following: abortion usually at the third trimester, retained placenta, stillborn, weak neonates, epididymitis, orchitis, reproductive failure and arthritis (hygroma).

*Probable brucellosis (Human):* A suspected case that has symptoms compatible with disease and is positive by Rose Bengal Test or quantitative polymerase chain reaction (qPCR).

*Probable brucellosis (Animal):* A case meeting the suspected case definition and/or postmortem findings and positive by Rose Bengal Test (RBT) in serum and milk ring test (MRT) in milk or qPCR.

*Confirmed brucellosis (Human):* Detection of *Brucella* spp. in a human sample by culture or demonstration of a 4-fold rise in titers in convalescent sera by Enzyme-Linked Immunosorbent Assay (ELISA).

*Confirmed brucellosis (Animal):* Positive serological results on compliment fixation test (CFT) or ELISA, or detection of *Brucella* spp. in animal sample by culture.

*Fora*—temporary satellite nomadic camp/dwelling unit established by pastoralists, usually in distant grazing lands away from the farmer’s permanent home/village, used during drought to reduce the distance between searching pasture and water for livestock and human consumption.

*Laga*—a man-made watering point or shallow well usually within the track or base of a seasonal river where pastoralists, their families and livestock access water. Also refers to the track/bed of a seasonal river.

*Manyatta—*a traditional hut made from sticks and grass or other locally available material, used by the Maasai and Samburu communities in East Africa. Also refers to a group of huts or compounds established within a common fence by a family or clan.

*Household—*A person or group of people who acknowledge one adult member as the head of the household, and who have common cooking arrangements. They may be related or unrelated but usually live together.

### 2.5. Sample Size

The sample size was determined using the Fleiss formula for cohort studies as illustrated below [38].

$$n^{\prime }=\frac{{\left[Z_{\alpha }\;\sqrt{\left(1+1/m\right)\bar{p}\left(1-\bar{p}\right)}+Z_{\beta \;}\sqrt{p_{0\;}\left(1-p_{0}\right)m+p_{1\;}(1-p_{1})}\right]}^{2}}{{\left(p_{0\;}-p_{1}\right)}^{2}}$$

$$\bar{p}=\frac{p_{1}+mp_{0}}{m+1}$$

$$n=\frac{n^{\prime }}{4}{\left[1+\sqrt{1\frac{2\left(m+1\right)}{nm|p_{0}-p_{1}|}}\right]}^{2}$$

where

n the estimated sample size per group

$p_{0}$ incidence of brucellosis among participants without camel exposure (unexposed)

$p_{1}$ incidence of brucellosis among participants with camel exposure (exposed)

$n^{\prime }$ sample size among exposed before continuity correction. The continuity correction brings normal curve probability in closer agreement with binomial probabilities.

m number of unexposed individuals per exposed individual

$\bar{p}$ estimated average of $p_{0}$ and $p_{1}$

$Z_{\alpha }$ the Z value corresponding to the alpha error of 5%. The corresponding (two-tailed) Z value is 1.96.

$Z_{\beta }$ the Z value corresponding to the beta error. The Z-value used was 0.80.

The sample size was calculated for human participants, assuming an average household size of 5 individuals, an incidence of brucellosis of 2% to 6% among the unexposed (26), detection of a risk ratio of at least 3 with a power of 0.80 and a two-sided alpha of 0.05. The incidence assumptions are supported by previous studies in similar pastoral populations. Munyua et al., 2021 [26] reported 2% annual incidence in pastoral communities in Kenya, while Cash-Goldwasser et al., 2018 [39] reported 9% incidence in northern Tanzania pastoral communities. The risk ratio of 3 is justified by data from Osoro et al., 2015 [28], demonstrating 2.5-fold higher household prevalence and 4-fold higher individual prevalence in camel-rearing versus non-camel-rearing communities in the same study counties.

Further accounting for the loss to follow-up of 30%, design effect of 1.5, and ability to conduct effective and efficient follow-up, a sample size of 170 households in each site was used. All eligible household members were targeted for enrollment. In each household, up to six animals per species (camels, cattle, goats, sheep) were selected at enrolment to identify up to three seronegative animals for enrollment and follow-up in the longitudinal cohort study. The decision was based on the observed 20% seroprevalence in our previous studies [23,28], with a preference for younger animals. To increase the probability of collecting samples from seropositive animals and enhance the chances of *Brucella* spp. isolation, up to five pregnant and five lactating animals per species were included in the longitudinal component (Table 1). The lactating animals were those whose milk is consumed or sold by the household. Preference was given to seropositive lactating livestock to increase the potential for yield of *Brucella* spp. Primary brucellosis infection in pregnant female livestock is a cause of late-pregnancy abortions [40].

The brucellosis infection then becomes chronic with subsequent birthing events associated with the shedding of the bacteria [40,41]. Pregnant livestock that were seropositive for brucellosis were enrolled for sampling during delivery or abortion events, including serum and vaginal swabs.

## 3. Study Procedures

The study was activated in November 2022 upon receiving the required ethical and administrative approvals. Presently, study field data collection has been finalized while laboratory analysis, data cleaning, data analysis and manuscript development are underway.

### 3.1. Community Engagement

Community engagement began with mapping potential study sites in the Marsabit and Kajiado counties, including assessments of local health facilities for essential infrastructure and consultations with local health and community leaders to guide study design and execution. These included the county and subcounty directors of health and veterinary services, county directors of environment and climate change, community health strategy focal persons, public health officers, disease surveillance coordinators, county commissioners, ward representatives, local administrators and community-owned resource persons such as respected elders, community health providers and community disease reporters. This comprehensive stakeholder engagement approach was essential to ensure the cultural appropriateness of study procedures, enhance community acceptance, and optimize the validity of our findings through incorporation of local knowledge about livestock management practices and brucellosis risk factors.

### 3.2. Baseline Cross-Sectional Survey

The baseline survey aimed to identify participants for the prospective longitudinal study and quantify the burden of brucellosis. This involved the identification of eligible households, screening and enrollment of eligible humans and livestock, collection of baseline biological samples and administration of baseline questionnaires for the household head, and individual livestock and human participants. All study questionnaires were previously standardized for use in our previous study in the same population [28]. In addition, a one-week study pilot was conducted, where the data collection tools, sampling procedures and sample chain of custody, study processes and team dynamics were tested and any necessary adjustments were made prior to implementation of the main study. The survey was conducted in November and December 2022 in Marsabit and in July 2023 in Kajiado. The respective human and animal samples were tested by appropriate ELISAs to determine baseline brucellosis serostatus. To ensure transparency, reproducibility, and contextualization of the data collection process, we have included complete versions of all the questionnaires used in this study as Supplementary files (q.v.)—S1: Study site characteristics; S2: Household baseline and follow-up questionnaires; S3: Herd baseline, follow-up, and clinical visit questionnaires; S4: Sick visit questionnaire; S5: Human–animal contact questionnaire; and S6: Laboratory protocols.

#### 3.2.1. Selection of Households and Herds

This study employed probability-proportional-to-size (PPS) sampling to select eligible households in 40 manyattas from seven villages in Marsabit and 71 manyattas from eight villages in Kajiado counties, respectively. We created a detailed list of villages and households within 30 km of the study reference health facility, each maintaining at least one livestock species. For each village, the number of households selected was distributed proportionally based on the relative human and livestock population and total number of households in that village. In both sites, households are clustered into compounds or dwelling units called *manyattas*. Within each selected manyatta/cluster, systematic random sampling was applied: the total number of livestock-keeping households was divided by the total number of sample households required to obtain the sampling interval. A random number was then determined for the starting point within the first sampling interval with subsequent household selections based on the sampling interval. We employed this multi-stage sampling approach to give representative coverage of the diverse pastoralist communities while maintaining statistical efficiency. The 30 km radius criterion balanced comprehensive geographical coverage with logistical feasibility for regular follow-up visits in challenging field conditions.

#### 3.2.2. Enrollment of Human Participants in Households

Trained study staff invited selected households to participate, obtaining consent from the household head or an eligible adult. Once consented, all household residents were listed to identify eligible participants for the study, with revisits scheduled, if necessary, within two weeks of the household enrolment. At the enrolment visit, standardized questionnaires on household socio-demographics and individual medical history, potential exposure to brucellosis, history and frequency of contact with livestock, signs and symptoms of acute brucellosis, health-seeking behavior and any treatment history in the preceding 12 months were administered and baseline blood samples collected. To allow for harvesting of at least 1 mL of serum from children and 3 mL from adults, approximately 5 mL of blood was collected from adults and children aged 13 years or older; 2–3 mL for children 5–12 years; and 1.5 mL for children < 5 years. These volumes were deemed safe [42] and adequate for initial testing and for storage for any subsequent testing accordingly. Finally, each household received an enrollment card with a toll-free contact number for reporting symptoms of brucellosis as well as any abortion events or illnesses in the herd.

#### 3.2.3. Enrollment of Livestock

In each enrolled household, we sampled livestock, including camels, cattle, goats, and sheep, representing various reproductive statuses such as pregnant, lactating, and non-pregnant/non-lactating individuals from each species. The protocol involved the collection of approximately 10 mL of blood from adult animals and 5 mL from young animals for *Brucella* spp. testing. In each enrolled household, animal and human participants were assigned a unique alpha-numeric study identification (ID) number for confidentiality and ease of identification during follow-up visits. Each enrolled animal was fitted with an ear tag. This unique ear-tag number is linked to the individual animal’s unique study ID number within the study database to facilitate identification during subsequent follow-up visits. All study samples were labeled immediately after collection with unique bar-coded labels and linked to an individual participant’s unique study ID, or ear-tag number in the case of animals. Data collected from enrolled animals included locality and morphometric data such as taxonomic name, age, and sex. Data on productivity, reproduction, breed, health status and signs and symptoms of suspected brucellosis were also recorded. In addition, any other livestock in the herd experiencing abortion events during the study period are purposively enrolled consecutively with the collection of vaginal and placental swabs. Data on herd demographic characteristics, livestock husbandry practices, and geospatial positioning (GPS and description) were also collected.

### 3.3. Prospective Longitudinal Cohort Study in Humans and Livestock

Enrolled human and animal participants were followed up every three months for 12–18 months, with the required data and specimens collected accordingly.

#### 3.3.1. Household and Participant Follow-Up Procedures

**Figure 4:**
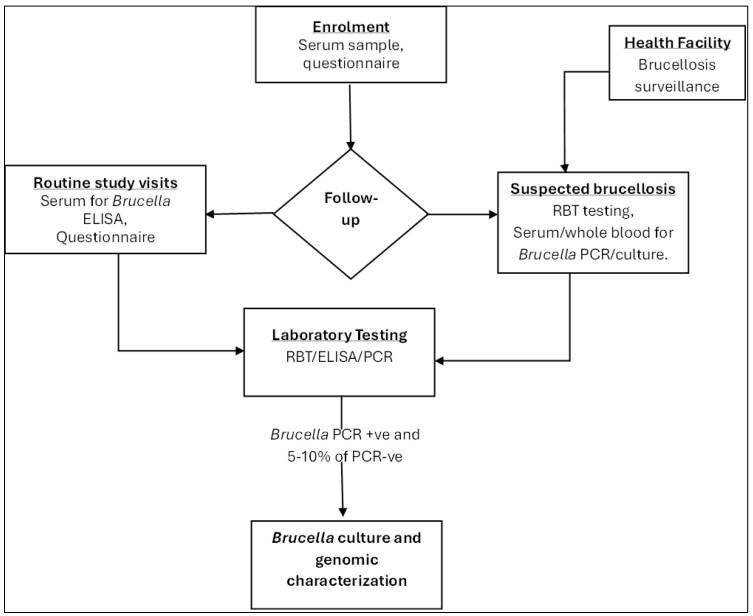
Summary of procedures for human participants. RBT—Rose Bengal Test, ELISA—Enzyme-Linked Immunosorbent Assay, PCR—Polymerase Chain Reaction, +ve (positive), −ve (negative).

At each follow-up visit, we collected blood samples from each human participant for *Brucella* spp. testing, following the same volume guidelines as at enrollment, and administered a structured follow-up questionnaire to gather information on exposure to brucellosis and any changes in medical history since the last visit (Figure 4). In cases where a participant was not present at their household during a visit, our study staff made additional attempts to conduct the procedures within the following seven days. Each household received livestock health incentives, including albendazole-based dewormers (active ingredient: albendazole 10%), mineral saltlicks containing essential trace elements (copper, zinc, selenium), cypermethrin-based acaricides for tick control, and KES 1500 (approximately USD 12) to offset participation costs.

#### 3.3.2. Livestock Follow-Up

**Figure 5:**
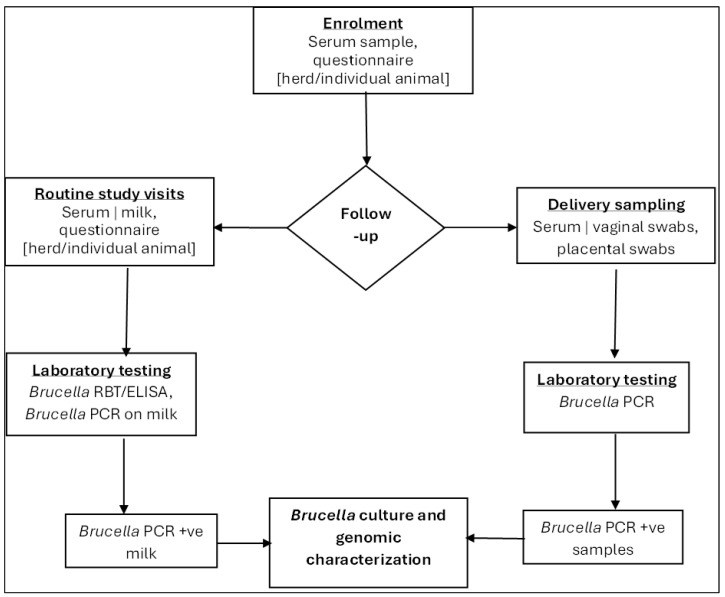
Summary of livestock follow-up procedures. RBT—Rose Bengal Test, ELISA—Enzyme-Linked Immunosorbent Assay, PCR—Polymerase Chain Reaction, +ve (positive).

At each three-month follow-up visit, the following samples were collected as shown in Figure 5.

- Serum samples from livestock determined seronegative by ELISA were collected at each follow-up visit for incident *Brucella* spp. infection detection. Sample collection used the same venipuncture protocols and serum separation procedures as baseline sampling (detailed in Supplementary Material S1).
- Milk samples (5–10 mL) were collected aseptically from all functional quarters of lactating animals using sterile falcon tubes following standard veterinary collection procedures.
- Vaginal swabs were collected using sterile Copan eSwab^®^ collection systems (Copan Diagnostics, Murrieta, CA, USA), through gentle insertion and rotation within the vaginal canal, with immediate placement in preservation medium. Collection occurred within 24 h of parturition or abortion events when feasible, with samples refrigerated immediately at 2–8 °C and transported to field laboratories within 6 h. Processing involved duplicate aliquot preparation for PCR and culture analysis, with detailed protocols following established veterinary diagnostic procedures for reproductive tract sampling.

We also administered a standardized routine visit herd and individual animal questionnaire.

#### 3.3.3. Monthly Telephone Calls

During the intervals between the three-monthly household visits, study staff made phone calls to collect health/clinical information on household members and enrolled animals. The window for the monthly calls opened at least two weeks after and closed at least two weeks before a routine household visit. Any reported abortion events in the herds led to a clinical visit for data collection, including vaginal swabs and blood samples. Participants reporting symptoms of acute febrile illness or suspected brucellosis during these calls were referred to the study health facility for evaluation and management by a clinician and subsequent enrollment into the study if eligible.

### 3.4. Health Facility Surveillance for Brucellosis

We prospectively recruited patients from household cohorts and others attending the study health facility who exhibited symptoms of suspected brucellosis. Those who met the study case definition and provided informed consent, assent and/or parental permission were enrolled for brucellosis screening using RBT at the health facility. RBT results were shared with the attending clinicians at the facility to guide the clinical care of participants based on Ministry of Health (MoH) guidelines.

Participants presenting with suspected brucellosis were interviewed using standardized sick visit questionnaires to collect clinical and exposure history data. The structured interview protocols captured symptom onset, duration, severity, and associated epidemiological risk factors relevant to brucellosis transmission dynamics.

Whole blood venous samples were also collected for RBT, ELISA, PCR, and *Brucella* culture. Quality assurance procedures included systematic processing of every tenth participant sample regardless of initial test results, standardized blood volume collection protocols across study sites, and comprehensive documentation requirements for clinical specimen handling. These procedures ensure methodological consistency and compliance with good clinical practice standards throughout the study implementation period. Participants with probable brucellosis were asked to provide a convalescent serum specimen after 4–6 weeks. Additionally, medical records of enrolled participants were reviewed and abstracted by study staff to collect information on diagnoses, test results, medical procedures, and hospitalizations during the follow-up period for the participants. The health facility surveillance was conducted concurrently with and throughout the community/household surveillance.

### 3.5. Laboratory Studies Including Molecular Typing

Table 2 presents the respective samples collected in this study and their subsequent chain of custody. Briefly, samples collected from humans and animals at the household were transported in separate cool boxes at 2–8 °C to the study field laboratory, where blood was centrifuged for serum separation. The serum, whole blood, vaginal swabs, and milk samples were stored at −20 °C for up to 72 h before shipment to the Kenya Medical Research Institute (KEMRI) Sample Management and Repository Facility (SMRF) in Nairobi where they were preserved at −80 °C until testing. All samples were shipped following standard shipment protocols [43].

#### 3.5.1. Rose Bengal Test (RBT)

RBT detects the presence of IgM, IgG and IgA antibodies against smooth *Brucella* spp. (*B. abortus*, *B. melitensis*, *B. suis*). The test utilized buffered *B. abortus* antigen supplied by the Animal and Plant Health Agency Scientific (APHA Scientific, New Haw, Addlestone, Surrey, UK), following modified RBT protocols previously validated in East African populations [44]. RBT is a simple, rapid slide-type agglutination assay performed with an antigen suspension of inactivated *B. abortus* stained with Rose Bengal stain. Agglutination indicates the presence of antibody in the test serum. Positive results were defined as visible agglutination within the observation period. The modified Rose Bengal Test employed in our study has demonstrated a sensitivity of 87.5% and specificity of 98.6% when used for human brucellosis in clinical settings [45,46].

#### 3.5.2. Enzyme-Linked Immunosorbent Assay (ELISA)

Human and animal sera were tested using validated ELISA: IBL *Brucella* IgG ELISA kits (Minneapolis, MN, USA) for human samples; and SVANOVIR^®^ *Brucella*-IgG Competitive-ELISA for livestock samples (Svanova Biotech AB, Uppsala, Sweden). The SVANOVIR^®^ *Brucella*-IgG ELISAs demonstrate documented sensitivity of 96–98% and specificity of 98–99% depending on the host species [47,48].

#### 3.5.3. *Brucella* spp. Quantitative PCR (qPCR)

Quantitative PCR will be conducted on blood samples from human participants with probable brucellosis as well as milk, vaginal swabs, and placental swabs from livestock in which brucellosis had been serologically confirmed. In addition, 10% of serologically negative animals will be included. Published protocols for the detection of *Brucella* species by qPCR will be employed, using the *bcsp31* gene and *IS711* insertion element targets, e.g., [49]. These qPCR assays detect all *Brucella* species, including vaccine strains.

#### 3.5.4. *Brucella* Culture

All *Brucella* qPCR positive samples, samples from probable brucellosis cases and 10% of qPCR negative baseline samples will be shipped to the WOAH/FAO Reference Laboratory for Brucellosis at the Animal and Plant Health Agency (APHA) in the United Kingdom for bacterial culture for *Brucella* spp. Specimen shipment to APHA (UK) will be performed according to international regulations for the transport of biological materials. Briefly, biological samples (whole blood and blood clots collected from humans and vaginal swabs, and milk collected from livestock), were preserved at –80 °C at the Kenya Medical Research Institute’s Sample Management and Repository Facility (SMRF) and shipped from Kenya to the Animal and Plant Health Agency (APHA) laboratory in the United Kingdom. Samples were packaged following International Air Transport Association (IATA) regulations for biological substances (UN 3373, Category B), with triple packaging, temperature loggers, and shipment under dry ice. All transfers maintained a documented chain of custody and complied with national and international biosafety, permitting, and material transfer regulations [50].

Human blood culture will be performed at the APHA (UK), using established protocols [37]. Two approaches will be used to culture human blood.

1. Inoculation of an appropriate non-selective liquid media, such as Tryptic Soy Broth (TSB), with subsequent plating/subculture at weekly intervals onto selective media such as Farrell’s media for up to 4 weeks.
2. Inoculation of Castañeda’s biphasic media which contains both a liquid and solid fraction.

Livestock milk, vaginal swabs and placental swabs will be cultured using both selective (Farrell’s) and non-selective (SDA) solid media. Additionally, liquid culture as described above will be performed. Abortion material will be macerated and then treated as described above. In animals, culture of milk, vaginal discharges, placental and fetal tissue is recommended, where such samples are available, as bacteremia in livestock is considered to be brief or intermittent. Pure cultures will be confirmed as members of the genus *Brucella* by qPCR targeting the *Brucella* specific insertion element *IS711* and *bcsp31* targets, as described above.

#### 3.5.5. Molecular and Genetic Characterization of *Brucella* spp.

We will perform multi-locus variable copy numbers of tandem repeats analysis (MLVA) and whole-genome sequencing (WGS) on all *Brucella* cultures isolated from animal and human samples. MLVA is likely to be the most informative approach with which transmission patterns of *Brucella* within and between the livestock and human populations of interest can be investigated [51]. The method is of particular value for discriminating between isolates in highly conserved bacterial species and genera, such as *Brucella*, and has consequently been widely used in the regional epidemiology of brucellosis infections. MLVA typing will be performed according to published protocols [52]. In addition to MLVA, we will perform WGS to further determine transmission pathways of particular *Brucella* spp. across the animal species and between animals and humans in a household/herd. WGS data for all isolates will be generated using established methods (Illumina short-read sequencing) [53]. Single nucleotide polymorphisms (SNPs) will be identified in the sequenced genomes using bioinformatics software for variant calling [54]. SNPs will then be used to undertake phylogenetic analyses to identify relationships amongst circulating *Brucella* isolates.

### 3.6. Contact Network for Human–Animal and Animal–Animal Interactions

#### 3.6.1. Human–Animal Contact Surveys

The contact network surveys were conducted during each study visit among all enrolled participants. Participants aged 13 years and above were asked to individually respond to the interviewer-administered contact questionnaire and those <13 years had the household head or other competent adult respond to the questions as a proxy. The questionnaire recorded the nature, number, duration, and frequency of contacts with livestock and their products within seven days preceding the interview. Contacts relevant to transmission of brucellosis that were investigated include consumption of raw milk or meat by livestock species, milking, feeding, slaughtering, and herding of livestock, butchering, assisting in birthing and removal of retained placentas, contact and handling of sick animals, contact with an aborted fetus, and handling animal hides and skins.

Evaluation of human–animal contact patterns, integrated with molecular epidemiological data, enables attribution of *Brucella* spp. infections to specific livestock species and transmission pathways. This analytical approach provides empirical foundations for evidence-based public health interventions targeting high-risk exposure scenarios in pastoral communities.

#### 3.6.2. Livestock Movements and Contacts Monitoring

To monitor livestock movement patterns, we fitted selected animals, representative of various herds and grazing patterns, with GPS loggers to longitudinally record their location and movement data. Livestock of different ages and sex were herded together either in nearby (usually within 10 km) or distant grazing fields. We selected one animal per species (regardless of *Brucella* test status) among those that graze within the village and another animal per species among livestock that are herded further away from the village. The livestock to be collared was identified by the household head as the one that represented or guided the general movement of the species or herd. We applied GPS receiver collars to the selected animals and allowed it to stay on for the 12–18 months study follow-up period. Device distribution occurred through collaboration with county and subcounty directors of health and veterinary services, community health strategy focal persons, ward representatives, community elders, and community disease reporters who provided local knowledge and facilitated community acceptance of monitoring technologies. The devices were distributed to ensure at least one device for every four households.

Livestock movement monitoring employed two GPS tracking systems: Savannah-type real-time satellite-enabled devices (Savannah Tracking Ltd., Kilifi, Kenya)—twenty devices; and CatLog2 GPS data loggers (Mr. Lee Catlog, Japan—http://www.mr-lee.com/catlog.htm (accessed on 15 March 2023))—sixty-two devices. Collar application was performed by trained veterinary personnel using traditional pastoral restraint methods, with appropriate sizing to prevent movement interference and regular monitoring protocols for animal welfare compliance. The GPS receiver recorded waypoints several times a day. Additionally, we collected GPS waypoints of the communal watering points to quantify the frequency of visits of enrolled herds to watering points and likelihood of inter-herd interactions at these communal gathering spots [55]. Eventually, we recorded the aggregate animal movement distances and quantified the frequency of contact among collared animals of different species. This livestock movement data will be applied in a dynamic disease model that includes the normalized difference vegetation index (NDVI) and climate variability data to inform effective prevention and control of brucellosis and other zoonotic diseases.

### 3.7. Ethnographic Studies on Human–Animal Interactions

The ethnographic component of the study relied on participant observation, where researchers embedded themselves within communities for three months. This involved living within and interacting naturally with the study community to gain a deep, insider’s perspective on their culture and practices. This immersion allowed them to build trust, observe and participate in daily lives of study participants, thereby understanding the nuances of the community’s social interactions and beliefs. At the same time, they observed and recorded high-risk practices for disease transmission, such as informal veterinary care and the management of animal by-products. The frequency and timings of participant observations relied on the actual events, practices, and social interactions known to influence brucellosis transmission risk, such as assisting in parturition, milking, slaughtering, unsafe dietary and sanitary practices, handling of afterbirths, handling of aborted fetuses and handling of skins and hides. These observations, backed by photographs and notes, will help to construct a comprehensive picture of community interactions with livestock from a socio-cultural and ecological viewpoint.

To supplement these observations, we conducted 60 individual in-depth interviews (IDIs) and 20 focus group discussions (FGDs) across study sites. These conversations included a diverse cross-section of community members to ensure a well-rounded perspective on local practices and knowledge relating to zoonosis. Additionally, 22 key informant interviews (KIIs) were completed to give deeper insights into community attitudes and practices, thus enriching our understanding of human–livestock dynamics. All these qualitative methods combined will contribute to a nuanced understanding of the factors influencing zoonotic disease transmission in pastoral settings.

Ethnographic findings will inform interventions through three pathways: (1) identification of high-risk cultural practices for incorporation into behavioral risk models and transmission parameter estimation, (2) development of culturally appropriate intervention designs that align with existing livestock management practices and community beliefs, and (3) formulation of community engagement strategies that leverage traditional leadership structures and communication channels identified through participant observation and key informant interviews.

### 3.8. Ethical and Regulatory Considerations, Informed Consent and Good Clinical Practice (GCP)

The study protocol was reviewed and approved by the KEMRI Scientific and Ethics Review Unit (reference # SERU 4405), the KEMRI Animal Care and Use Committee (reference # KEMRI/ACUC/02.07.2022), the Washington State University Institutional Animal Care and Use Committee (reference # ASAF 7081). Appropriate administrative approvals will be sought from the Ministry of Agriculture, Livestock and Fisheries, Ministry of Health, National Commission for Science Technology, and Innovations (License No: NACOSTI/P/22/17621) and the Director General for Health, the Director for Veterinary Services, and the respective county governments. In addition, the US Centers for Disease Control and Prevention reviewed and approved the study protocol (CDC Reliance # 7458)

The household head or designee provided written consent for their livestock and household members to participate, while all participants individually provided written consent, assent and/or parental permission before any study procedures could be undertaken. At household level, detection of seropositive lactating and pregnant animals will be shared, along with information on brucellosis risk reduction measures. Any information obtained from this study that might be important for the health and welfare of the livestock owner or his/her family, especially about zoonoses, will be communicated through the County Veterinary Office.

The study employs the participatory community engagement model for initial and ongoing meaningful engagement of all stakeholders, including community stakeholders, to include sensitization, education and ongoing feedback [56]. The participatory model briefly espouses communication, consultation and collaboration throughout and beyond the project lifecycle.

Any human participant requiring medical care is referred to the clinical team at the study facility or other health facilities in the study area for evaluation and appropriate management. All livestock are manually restrained or restrained within a crush for sample collection with every care taken to minimize sampling-associated stress to the animals. In the event of human injury during animal sampling (including all project personnel and household members participating in interviews/sampling), appropriate first aid is provided by study staff, and the injured person would be immediately taken to a medical facility for further treatment, if needed.

*Sharing of test results***.** Results of brucellosis-positive animals are shared with the livestock owners through the county veterinary department and information regarding prevention of transmission from animals to humans is reinforced. Active surveillance for acute human brucellosis will be performed real-time, and results shared with attending clinician for clinical care according to MoH guidelines.

### 3.9. Data Management and Analysis

Quantitative data were collected electronically using tablets through the REDCap mobile application (Vanderbilt University Medical Center, Nashville, TN, USA), which provides encryption at rest and during data transmission. All study data in the tablets were synced to the central database using a secure link. Data were checked routinely through high-frequency data checks for completeness, accuracy, and consistency.

For qualitative data management and field level analysis, each week of fieldwork was followed by a buffer day—a day when research assistants are expected to translate the notes into English, transcribe, type them, and check for clarity and completeness of the verbatim notes, before submitting them to the lead ethnographer. Once transcription was complete, the researchers read while re-listening to correct any errors, anonymize participants, insert notations, punctuation and contextual information that might have affected the participant. All the audio-recordings were transcribed verbatim. All the qualitative data (field notes and expanded notes, photographs, audio files and transcripts) were stored in password-protected data storage hosted on the Washington State University secure server.

Qualitative analysis involved debriefing sessions at every fieldwork day to obtain a summary of key and emerging issues in FGD, KII and IDI conversations. These exercises help to establish the emerging themes of the study following collective insights on the value and nature of observed behaviors including reflections on photographs taken. Subsequently, NVivo 14 (QSR International Pty Ltd. Lumivero, Denver, CO, USA), will be used to organize, code, and disaggregate the textual material for qualitative analysis. Data will be analyzed deductively. By prospectively following up individuals and their livestock with periodic data and sample collection, our study will identify and track the spread of new brucellosis infections and show the interplay and utility of molecular data alongside interconnected social networks and geospatial systems. R Software version 4.3.3 (R Foundation for Statistical Computing, Vienna, Austria) will be used to analyze quantitative data.

Several quality assurance measures will be implemented as part of this study. These include standardized training of all field study staff, development of structured data collection instruments with pre-validated fields, and methodological triangulation through multiple data sources (questionnaires, observational data, and biological specimens). Research assistants were recruited from local communities to enhance data validity by providing essential linguistic and cultural context during data collection. In addition, the use of electronic data capture through REDCap with built-in validation algorithms, daily data synchronization, and automated quality checks was to minimize entry errors.

Missing data patterns will be systematically assessed to determine mechanisms (missing completely at random, missing at random, or missing not at random). Multiple imputation will be considered for key covariates with missingness >25%, while inverse probability weighting will be applied to adjust for differential loss patterns by exposure status. Sensitivity analyses will compare complete case analysis with imputed datasets to assess robustness of findings. For livestock losses due to death or sale, survival analysis techniques will account for informative censoring.

The primary outcomes will be (1) the detection of *Brucella* spp. in humans and animals and (2) the incidence rate of brucellosis among humans and livestock, defined as the detection of *Brucella* spp. in a human or animal sample by culture or by demonstration of a 4-fold rise in anti-*Brucella* antibody titers in convalescent human sera compared with the person- or livestock-follow-up time. The incidence rate will be adjusted for clustering and factors such as age and herd size.

**Figure 6:**
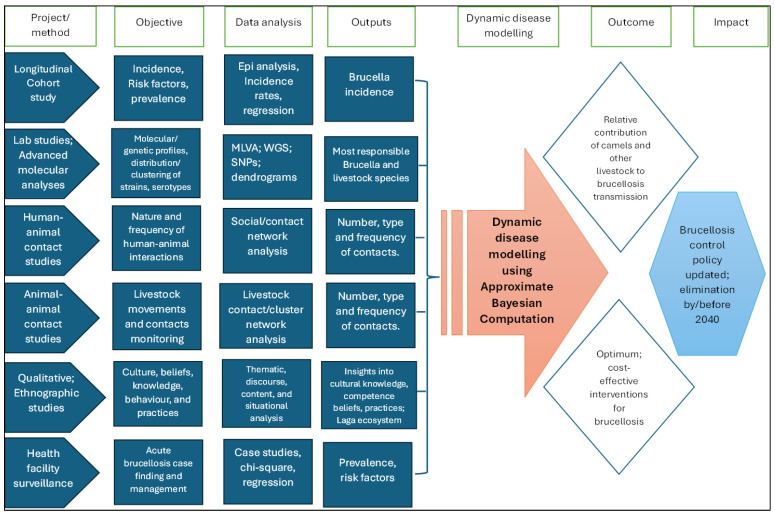
Design-, data analysis- and disease-modeling approach for the transdisciplinary, multi-method brucellosis research project. Epi—epidemiological analysis; MLVA—multiple locus variable number of tandem repeats analysis; SNPs—single-nucleotide polymorphisms; Spp.—species; WGS—whole-genome sequencing.

We will use data from the cohort and contact network studies (Figure 6), to parameterize a multi-species mathematical model of brucellosis transmission to disentangle the contributions of the different livestock species to *Brucella* maintenance and transmission. Specifically, we will use Approximate Bayesian Computation [57] to parameterize the multi-species model using study data from the longitudinal cohort and contact network studies. ABC works by drawing model parameters at random from a prior distribution, using the model to simulate a dataset, and adding the randomly selected parameters to the posterior distribution. Repeating this process leads to an approximate solution for the posterior distribution. We will use the resulting posterior distribution to calculate and compare the force of infection from each animal species into the human population. The model will break the animal population down into classes, tracking how the number of susceptible (S), infected (I), and immune (R) animals change over time. We will generalize and extend these models to include multiple livestock reservoir species and humans, as well as both *B. abortus* and *B. melitensis*. The result will be a system of equations describing epidemiological dynamics of *Brucella* within livestock species and spillover into the human population. We will then use the parameterized model to quantify the contributions of each livestock species to *Brucella* maintenance and to predict within and between force of infection from each animal species and into the human population (Figure 6).

We will then draw parameters at random from the posterior distribution and implement in silico interventions using stochastic simulations of the model. These simulated interventions naturally integrate the complex network of transmission among livestock species and provide concrete quantitative estimates for the outcome of interventions and the timescale over which reductions in human infection can be expected to occur. Thus, the model will capture transmission of *Brucella* strains within and among livestock species, as well as the transmission of *Brucella* from each of these species into the human population, allowing for estimation of strain and species-specific transmission rates.

To assess the robustness of our findings, we will conduct sensitivity analyses by systematically varying key model parameters (incidence rates, dropout rates, and diagnostic test performance) within plausible ranges. This will allow us to determine threshold values at which study conclusions might change and quantify the uncertainty in our estimates. The ABC framework inherently supports this sensitivity analysis by generating posterior distributions that reflect parameter uncertainty rather than point estimates, which are particularly valuable given the environmental and logistical variability in pastoralist communities.

### 3.10. Data Integration and Analytical Framework

The study employs a sequential mixed-methods approach for data integration. Quantitative data (serological, molecular, GPS tracking) will be analyzed using R Software to determine transmission parameters and contact networks. Qualitative ethnographic data will be coded using NVivo 14 and analyzed thematically to identify behavioral risk factors and cultural practices influencing transmission. Integration occurs through (1) triangulation of quantitative risk factors with qualitative behavioral patterns, (2) incorporation of ethnographic findings into contact network models, (3) parameterization of the multi-species transmission model using Approximate Bayesian Computation with combined datasets, and (4) development of culturally informed intervention strategies based on integrated findings. Figure 6 illustrates this transdisciplinary analytical framework, leading from field data collection to policy-relevant recommendations.

## 4. Expected Results and Discussion

This manuscript presents a study, rather than analytical findings or study results. Our study is poised to advance the understanding of the transmission dynamics of *Brucella* spp. in pastoralist settings. We anticipate novel insights into the role of camels, a largely underrepresented species in zoonotic disease research, in transmitting these pathogens to humans. This research is important for both arid and semi-arid regions where pastoral communities closely interact with livestock. Our findings are expected to inform more effective public health strategies and interventions, including the development of targeted surveillance programs, vaccination campaigns, and community education initiatives. These strategies will be specifically designed to address the risks associated with brucellosis in pastoralist settings, thereby enhancing their public health significance and potential for improving disease control and prevention.

Our study employs a methodological approach grounded in the One Health concept, recognizing the interconnectedness of human health, animal health, and their shared environment. Unlike previous brucellosis control models, this multidisciplinary framework, combining epidemiological analysis, molecular characterization, disease modeling, ethnographic data, and network analysis of animal movements, is essential for a comprehensive understanding of *Brucella* spp. transmission dynamics, optimized testing strategies and cost-effective elimination programs (Figure 6), that align with the national strategy for brucellosis control in Kenya by 2040 [31]. Also, due to adaptation to climate change, pastoralists in traditionally non-camel-rearing communities and regions have increasingly embraced camel farming with the attendant risk of emergence of camel-borne zoonotic diseases in these regions. Our model can predict and recommend appropriate responses to these potential threats before they emerge.

However, this methodological rigor comes with inherent challenges, such as the potential biases in self-reported data, failure or loss of GPS devices, and logistical difficulties in tracking animal movements across varied terrains. A particularly notable challenge is the loss of follow-up of enrolled animals, which could impact the study’s longitudinal analysis. Furthermore, the challenges of isolating *Brucella* spp., including its growth requirements and biosafety considerations, add another layer of complexity to our study. These factors may affect the interpretation and generalizability of our findings, particularly in the unique socio-ecological context of the Marsabit and Kajiado counties, Kenya. Despite these challenges, our comprehensive One Health approach is vital for developing effective interventions and ensures that our findings are relevant across the domains of medical and veterinary public health.

The findings from our study are anticipated to lay the groundwork for several areas of future research. One key area is further epidemiological studies to deepen understanding of the transmission of zoonotic pathogens in varied geographical and cultural contexts. Additionally, the development and investigation of targeted vaccination strategies for brucellosis, particularly suited for pastoralist communities, would be a valuable advance. Public health interventions, including educational programs and improved surveillance systems, as well as *Brucella* vaccine development, can also be refined and assessed based on our study outcomes. These research directions will be instrumental in advancing global efforts to control and prevent zoonotic diseases in diverse populations.

## 5. Conclusions

In conclusion, this study will significantly enhance our understanding and management of zoonotic diseases in pastoralist settings. By focusing on the transmission dynamics of *Brucella* spp. among camels and other livestock, it addresses a critical gap in current research. The potential contributions of our findings include enhancing livestock disease control strategies, informing public health interventions, and guiding future research. This work underscores the importance of addressing health challenges in pastoralist communities, which is crucial for national and regional efforts in controlling zoonotic diseases.

## Figures and Tables

**Table 1:** Summary of livestock numbers targeted for sampling by species.

Species	Primary Animals *	Pregnant Animals	Lactating Animals	Total Animals per Household	% of HHs with Livestock (75% of Total)	Total Animals
Camel	6	5	5	16	127.5	2040
Goat	6	5	3	14	127.5	1785
Sheep	6	5	3	14	127.5	1785
Cattle	6	5	5	16	127.5	2040
	Total					7650

* Primary animals were neither pregnant nor lactating; and included young or nulliparous animals, and male breeders; HH = Household.

**Table 2:** Summary of brucellosis sample collection, storage and testing procedures.

Sample Type	Diagnostic Tests	Collection Volume	Laboratory Processing	Storage	Testing Laboratory	Manufacturer Details
Serum (Human)	RBT, IgG ELISA	1–5 mL	Centrifugation at 3000 rpm, serum separation	−20 °C field, −80 °C KEMRI-SMRF	Study facility, KEMRI-CVR	IBL *Brucella* IgG ELISA (Minneapolis, MN, USA); APHA RBT antigen (Surrey, UK)
Serum (Animal)	RBT, Species-specific ELISA	5–10 mL	Centrifugation at 3000 rpm, serum separation	−20 °C field, −80 °C KEMRI-SMRF	KEMRI-CVR	SVANOVIR^®^ *Brucella*-IgG ELISA (Uppsala, Sweden); APHA RBT antigen (Surrey, UK)
Whole Blood	*Brucella* qPCR, Culture	1–5 mL (human), 5–10 mL (animal)	DNA extraction, culture preparation	−20 °C field, −80 °C KEMRI-SMRF	KEMRI-CVR, APHA (UK)	Applied Biosystems reagents; APHA culture protocols
Milk	*Brucella* qPCR, Culture	5–10 mL aseptically collected	Duplicate aliquots for molecular and culture analysis	−20 °C field, −80 °C KEMRI-SMRF	KEMRI-CVR, APHA (UK)	Applied Biosystems reagents; APHA culture protocols
Vaginal/Placental Swabs	*Brucella* qPCR, Culture	Copan eSwab^®^ collection system	Immediate preservation, duplicate processing	−20 °C field, −80 °C KEMRI-SMRF	KEMRI-CVR, APHA (UK)	Copan Diagnostics, Murrieta, CA, USA; Applied Biosystems, Waltham, MA, USA

Storage conditions: field −20 °C, long-term −80 °C at KEMRI-SMRF. APHA (UK): Animal and Plant Health Agency, UK; KEMRI-CVR: Kenya Medical Research Institute Centre for Virus Research; qPCR: Quantitative Polymerase Chain Reaction; QFU: quarterly follow-up survey; SMRF: Sample Management and Repository Facility; IATA: International Air Transport Association; mL: milliliter.

## Data Availability

Not applicable. Data sharing is not applicable to this article as no datasets were generated or analyzed during the current study.

## Supplementary Materials

The following supporting information can be downloaded at: https://www.mdpi.com/article/10.3390/ijerph22121859/s1. Supplementary Table S1: Study site characteristics. Supplementary Material S1: Laboratory protocols; Supplementary Material S2: Household baseline and follow-up questionnaires; Supplementary Material S3: Herd baseline, follow-up, and clinical visit questionnaires; Supplementary Material S4: Brucellosis sick visit questionnaire; Supplementary Material S5: Human–animal contact questionnaire.

## Abbreviations

The following abbreviations are used in this manuscript:

ABC	Approximate Bayesian Computation
ACUC	Animal Care and Use Committee
AFI	Acute Febrile Illness
APHA	Animal and Plant Health Agency in the United Kingdom
ASAL	Arid and Semi-arid Lands
ARI	Acute Respiratory Illness
BSL	Biosafety Level
CFT	Complement Fixation Test
DTRA	Defense Threat Reduction Agency
ELISA	Enzyme-Linked Immunosorbent Assay
GPS	Global Positioning System
IATA	International Air Transport Association
IPC	Infection Prevention and Control
KEMRI	Kenya Medical Research Institute
CVR	KEMRI’s Center for Virus Research
MLVA	Multiple Locus Variable Copy Numbers of Tandem Repeats Analysis
MoH	Ministry of Health
MRT	Milk Ring Test
NACOSTI	National Commission for Science, Technology and Innovation
REDCap	Research Electronic Data Capture
WSU GH	Washington State University Global Health
RBT	Rose Bengal Test
qPCR	Real-Time Quantitative Reverse Transcriptase Polymerase Chain Reaction
SERU	KEMRI’s Scientific and Ethics Review Unit
SMRF	Sample Management and Repository Facility
SNPs	Single-Nucleotide Polymorphisms
SOP	Standard Operating Procedure
Spp.	Species
PPE	Personal Protective Equipment
WOAH	World Organization for Animal Health
WGS	Whole-Genome Sequencing
WHO	World Health Organization
ZDU	Zoonotic Diseases Unit
