# Supplementary material for: Design of a Prospective Human–Animal Cohort Study to Evaluate the Role of Camels and Other Livestock Species in the Transmission of Brucella spp. to Humans in Kenya
Source: Int J Environ Res Public Health. 2025 Dec 12;22(12):1859. doi: 10.3390/ijerph22121859 (PMC12733042; doi:10.3390/ijerph22121859)
Supplement: Supplementary file 1 [file ijerph-22-01859-s001.zip › Supplementary Material S1_Laboratory Protocols.pdf]

Supplementary Material S1: Laboratory Protocols

Sample Collection Procedures. Blood collection from livestock involved jugular venipuncture using sterile BD Vacutainer® tubes without anticoagulant (red-top serum tubes) to facilitate serum separation. The samples were collected by a veterinary officer or veterinary technician after the animal was adequately restrained. Human blood samples were obtained through antecubital venipuncture following identical protocols. To facilitate safe, efficient and coordinated animal sampling, each sample (tube) was labelled immediately using an indelible/permanent marker pen (sharpie) the household and animal ID reflecting the location of the herd as well as the details of the individual animal species, sex and age and the ordered number for the sample. Upon completion of sampling for that household and before moving to the next household/herd, all samples were then systematically labelled with with unique bar-coded identifiers linked to individual study ID numbers to ensure accurate tracking throughout the analytical process. Transport conditions from collection to field storage maintained specimens at 2-8°C in insulated cool boxes equipped with ice packs and temperature monitoring devices. The maximum allowable transport time from collection to laboratory processing was established at 12 hours to preserve sample integrity and diagnostic accuracy.

Chain of Custody Protocols Field to laboratory transfer procedures required comprehensive documentation including sample manifests, temperature logs, and chain of custody forms signed by both field personnel and laboratory staff. Temperature monitoring during transport utilized calibrated data loggers with continuous recording capabilities to verify maintenance of cold-chain integrity. Sample integrity verification occurred upon receipt at laboratory facilities through visual inspection, temperature verification, and documentation of any anomalies.

Cold-Chain Contingency Measures. The Kenya Medical Research Institute's Sample Management and Repository Facility (KEMRI-SMRF), maintain 24-hour backup generator systems with automatic switchover capabilities to ensure uninterrupted cold storage during power failures.

Biosafety Protocols Sample processing occurs within enhanced BSL-2 facilities that implement BSL-3 practices for high-risk specimens, particularly placental materials and abortion-related samples. Duplicate sample storage in separate freezer units provides insurance against equipment failures while maintaining sample availability for confirmatory testing.
