# Supplementary material for: Design of a Prospective Human–Animal Cohort Study to Evaluate the Role of Camels and Other Livestock Species in the Transmission of Brucella spp. to Humans in Kenya
Source: Int J Environ Res Public Health. 2025 Dec 12;22(12):1859. doi: 10.3390/ijerph22121859 (PMC12733042; doi:10.3390/ijerph22121859)
Supplement: Supplementary file 1 [file ijerph-22-01859-s001.zip › Supplementary Material S2_Household Baseline and Follow-up Questionnaire.pdf]

Appendix 1: Enrolment Household and Individual Questionnaire

SECTION A. GENERAL INFORMATION [FILL IN CAPITAL LETTERS]

**A1. Compound head name:**			
**A2. Household head name:**			
**A3. Household ID (From list):**		**A4.Date of interview: (dd/mm/yy)**	
**A5. Telephone of household head:**			
**A6. Enumerator initials:**			

**A7. County**	1= Kajiado<br><br>2= Marsabit

MARK THE POINTS WITH HOUSEHOLD ID AND SAVE ON THE GPS RECEIVER

SECTION B. HOUSEHOLD DEMOGRAPHICS

ENTER INFORMATION IN TABLE BELOW ON ALL HOUSEHOLD MEMBERS CURRENTLY LIVING (WITHIN LAST WEEK) IN THE HOUSEHOLD.

**B1. Household Member Name.**	**B2. Position in the Household** (in relation to household head)	**B3. Age (years)**	**B4. Age in months(If less than 1 year)**	**B5. Sex**	**B6. Highest level of formal education completed**	**B7. Primary occupation (Pick one)**	**B8. Role (check all that apply)**

	1=Household Head<br>2=Spouse of Household Head<br>3=Son/Daughter<br>4=Sibling<br>5=Father/Mother<br>6=Nephew/niece<br>7=Grand (son/daughter)<br>8=Herdsman/woman<br>9= House help<br>10=Other (Specify above)	(Years)		1=Male<br>2=Female	0=No formal education<br>1 = Primary<br>2 = Secondary<br>3 = Post secondary<br>4-Adult education<br>5 = Other	1. Works on farm/Farmer<br>2. Salaried off farm non skilled<br>3. Salaried off farm skilled<br>4. Student<br>5. Housewife<br>6. Other(specify)	1=Milking<br>2=Slaughtering<br>3 =Butchering<br>4= Cleaning barns<br>5= assisting in animal delivery<br>6. = herding/<br>7. Feeding animals<br>8.=Other<br>9- Don't know

B9. Are there any livestock (cattle, goats, sheep, camels) in this household?

◯ Yes ◯ No

B10. Who usually makes decisions in the management (TREATMENT, GRAZING, SELLING) of animals? (TICK ONE)

1=Household Head
2=Spouse of Household Head
3=Son/Daughter
4=Sibling
5=Father/Mother
6=Nephew/niece

7=Grand (son/daughter)
8=Herdsman/woman
9=Other (Specify above)

SECTION C: *KNOWLEDGE ATTITUDES AND PRACTICES*

I would now like to ask you some questions on brucellosis

C1. Have you ever heard of brucellosis before today? IF NO SKIP TO C15

○ Yes ○ No ○ Don't know

C2. Are animals affected by brucellosis? IF NO, SKIP TO C8

○ Yes ○ No ○ Don't know

C3. Do you know which animals are affected by brucellosis? IF NO, SKIP TO C5

○ Yes ○ No ○ Don't know

C4. Which animals are affected by brucellosis? (DO NOT PROMPT. CHECK ALL THAT APPLY.)

☐ Cattle ☐ Goats ☐ Sheep ☐ Pigs
☐ Dogs ☐ Camels ☐ Other (specify) ________________________

C5. How is brucellosis spread among animals? (DO NOT PROMPT, TICK ALL THAT APPLY)

☐ Ingestion of contaminated food/pasture
☐ Drinking contaminated water
☐ Contact with wild animals
☐ Sexual
☐ Other (specify) ________________________________
☐ Don't know

C6. How is brucellosis prevented in animals? (DO NOT PROMPT. TICK ALL THAT APPLY)

☐ Vaccination ☐ Drug treatment ☐ Slaughtering
☐ Other (specify) ___________________________ ☐ Don't know

C7. How can you tell if an animal has brucellosis? (DO NOT PROMPT. TICK ALL THAT APPLY)

☐ Abortions ☐ Swollen joints ☐ Retained placenta ☐ Reduced milk production ☐ Swollen testes
☐ Infertility ☐ Others (specify)____ ☐ Don't know

C8. Are humans affected by brucellosis? IF NO OR DON'T KNOW, SKIP TO C15

◯ Yes ◯ No ◯ Don't Know.

C9. How do humans get brucellosis? (DO NOT PROMPT. TICK ALL THAT APPLY).

☐ Contact with aborted animal fetus
☐ Drinking/eating raw dairy products
☐ Slaughtering animals
☐ Drinking animal blood
☐ Eating uncooked/undercooked meat from an infected animal
☐ Milking
☐ Herding
☐ Sharing sleeping space with animals
☐ Other(specify)_______________________________________
☐ Don't know

C10. How can you tell that somebody has brucellosis? (DO NOT PROMPT, TICK ALL THAT APPLY).

☐ Hotness of body ☐ Chills ☐ Fatigue ☐ Lack of appetite ☐ Joint pains ☐ Abortions
☐ Headache ☐ Others (specify) _______________________________________ ☐ Don't know

C11. Do you know of anyone who has ever had brucellosis? IF NO, SKIP TO C13

◯ Yes ◯ No ◯ Don't know

C12. If yes, who? (DO NOT PROMPT, TICK ALL THAT APPLY).

☐ Family member ☐ Relative ☐ Friend
☐ Self ☐ Co-worker ☐ Other ________________________________

C13. Do you know how brucellosis is prevented in humans? IF NO OR DON'T KNOW, SKIP TO C15

◯ Yes ◯ No ◯ Don't know

C14. How is brucellosis prevented in humans?

☐ Vaccination ☐ Drug treatment ☐ Boiling milk ☐ Other (specify) ________________________

C15. How are aborted fetuses /placentas /still born from animals disposed off? (DO NOT PROMPT, TICK ALL THAT APPLY).

☐ Eaten by dogs ☐ Buried/thrown in pit latrine ☐ Left out in the pasture ☐ Eaten by humans ☐ Burnt

☐ Others (specify)____

C16. Where do you get milk/dairy products (yoghurt, cheese, mala) for domestic use? (DO NOT PROMPT, TICK ALL THAT APPLY).

- ☐ Milk/dairy products from own animals
- ☐ Milk/dairy products from neighbor
- ☐ Milk/dairy products from market/vendor
- ☐ Pasteurized milk/dairy products from shop
- ☐ Milk not consumed in this household (IF SELECTED SKIP TO C20)

C17. The milk consumed in the household is from which animals? (DO NOT PROMPT, TICK ALL THAT APPLY).

☐ Cattle ☐ Sheep ☐ Goats ☐ Camels ☐ Others (specify)___________________ ☐ Don't know

C18. Do you boil milk before drinking? ○ Yes ○ No ○ Don't know

C19. If yes how often do you boil milk before drinking? ○ Always ○ Sometimes ○ Don't know

C20. How is milk obtained from your animals utilized? (DO NOT PROMPT, TICK ALL THAT APPLY).

☐ Neighbourhood sales ☐ Sale in local market ☐ Feed young animals only ☐ Home Consumption

☐ Sale to local hotels ☐ Sale to cooperatives ☐ Do not have livestock ☐ Others

C21. In case of domestic consumption, how do you treat the milk? (DO NOT PROMPT, TICK ALL THAT APPLY).

☐ No Preservation ☐ Boiling ☐ Chilling ☐ Other Specify)____________________________

C22. Is the children's milk treated differently? IF NO SKIP TO C24 ◯Yes ◯No ◯Don't know

C23. If yes, how is it treated? (DO NOT PROMPT, TICK ALL THAT APPLY).
☐ Boiling ☐ Fermented ☐ Chilled ☐ Other (specify) ___________________________

C24. Do you mix milk from different animals within your herd? ◯Yes ◯ No ◯Don't know

C25. What milk products do you consume? (DO NOT PROMPT, TICK ALL THAT APPLY).
☐ Fresh Milk ☐ Pasteurized fermented milk ☐ Unpasteurized fermented milk Unpasteurized yoghurt
☐ Pasteurized (packaged) yog☐rt Unpasteurized cheese ☐
☐ Butter ☐ Pasteurized (packaged) cheese

C26. At what age are young children started on milk other than breast milk? TICK ALL THAT APPLY AND INDICATE THE AGE
☐ Cow milk __________months
☐ Goat milk __________months
☐ Camel milk _________ months
☐ Other milk type (specify) _________months

C27. Which milking methods/gadgets do you use?
☐Hand Milking ☐Machine Milking ☐Other (specify) _____________________________________

C28. Does anyone in your household assist animals during deliveries? ◯ Yes ◯ No ◯Don't know

C29. Who normally assists in the deliveries? DO NOT PROMPT, TICK ALL THAT APPLY
☐Father ☐ Mother ☐ Other family member ☐ Neighbor/relative ☐ Animal health official
☐ Other (specify)____________________________________

C30. Do they use gloves when assisting in animal deliveries? ◯ Yes ◯ No ◯ Don't know

C31. Have you ever received information on brucellosis from anyone before today? IF NO SKIP TO C33
Yes ◯ No ◯ Don't know ◯

C32. If Yes, from whom? (DO NOT PROMPT, TICK ALL THAT APPLY).
- ☐ Animal health workers
- ☐ Human health workers
- ☐ Posters
- ☐ Electronic media
- ☐ Print media
- ☐ Friends
- ☐ Religious leader
- ☐ Community meetings (baraza)
- ☐ Other (specify)____________________________________

C33. Where do you typically get health information? (DO NOT PROMPT, TICK ALL THAT APPLY).
☐ Health facility ☐ Mass media ☐ Community member
☐ Older adult in household ☐ Friends/Neighbors ☐ Extension worker ☐ Other (specify) ________________

C34. How would you like to receive health information? (DO NOT PROMPT, TICK ALL THAT APPLY).
☐ Health worker ☐ TV ☐ Radio ☐ Community member ☐ Family member ☐ Religious leader
☐ Print media ☐ Extension worker ☐ Other (specify) ____________________________________

PART II: HUMAN PARTICIPANT FORM

This will be administered to each individual enrolled

SECTION E: INDIVIDUAL DEMOGRAPHICS

E1. Household ID:________________

E2. Specimen ID __________________

E3. Date of collection (dd/mm/yy) ________________________

**E4. HHd Member No.**	**E5. Position in the Household** (in relation to household head)	**E6. Age (years)**	**E7. Age in months(If less than 1 year)**	**E8. Sex**	**E9. Highest level of formal education completed**	**E10. Primary occupation (Pick one)**	**E11. Role (check all that apply)**
	1=Household Head<br>2=Spouse of Household Head<br>3=Son/Daughter<br>4=Sibling<br>5=Father/Mother<br>6=Nephew/niece<br>7=Grand (son/daughter)<br>8=herdsman/woman<br>9=house help<br>10=Other (Specify above)	(Years)		1=Male<br>2=Female	0=No formal education<br>1 = Primary<br>2 = Secondary<br>3 = Post secondary<br>4-Adult education<br>5 = Other	1. Works on farm/Farmer<br>2. Salaried off farm non skilled<br>3. Salaried off farm skilled<br>4. Student<br>5. Housewife<br>6. Other(specify)	1=Milking<br>2=Slaughtering<br>3 =Butchering<br>4= Cleaning barns<br>5= assisting in animal delivery<br>6. = herding/<br>7. Feeding animals<br>8.=Other<br>9- Don't know

E12. Have you had any febrile illness in the past 12 months? IF NO OR DON'T REMEMBER SKIP TO G1

◯Yes ◯No ◯ Don't remember

E13. If yes, how many times? _______

E14. How long ago was the most recent febrile illness? ___________months (IF CURRENT FEBRILE ILLNESS INDICATE ZERO)

E15. Onset of most recent febrile illness

◯ Acute (sudden) ◯ Insidious (gradual) ◯ Cannot remember

E16. Duration of illness in days ___________

SECTION F: MEDICAL HISTORY

F1. What signs and symptoms did/ do you exhibit? DO NOT PROMPT. IF TICKED, INDICATE DURATION IN DAYS

- ☐ Hotness of body, Intermittent ……………………………….
- ☐ Hotness of body, Constant ……………………………….
- ☐ Chills ……………………………….
- ☐ Weight Loss ……………………………….
- ☐ Night Sweats ……………………………….
- ☐ Headache ……………………………….
- ☐ Malaise ……………………………….
- ☐ Lack of appetite ……………………………….
- ☐ Stiff or painful neck ……………………………….
- ☐ Joint or muscle pain ……………………………….
- ☐ Back pain ……………………………….
- ☐ Abdominal pain ………………………………….

F2. Have you had a spontaneous abortion (Miscarriage) (FOR FEMALES ABOVE 15 YEARS)

◯ Yes ◯ No ………………………………….

F3. If other symptom, specify …………………………………………………………………….

F4. Have you been treated for any febrile illness in the last 12 months? (IF NO SKIP TO G1)

◯ Yes ◯ No ◯ Unknown

F5. If yes, where were you treated?

- ☐ Public health facility
- ☐ local chemist
- ☐ traditional healer
- ☐ private clinic
- ☐ mobile clinic
- ☐ acquired medicine from
  - ☐ Shop
  - ☐ Friend
  - ☐ Relative
  - ☐ Self medication with Herbs
  - ☐ Other (specify) ________________________________

F6. What medicines did you take?

- ☐ Antibiotics
- ☐ Antimalarial
- ☐ Pain killers
- ☐ Herbal
- ☐ Other (specify) _______________________________________
- ☐ Don't know

F7. For how long did you take the medication? NUMBER OF DAYS…__________………

F8. How many times have you been treated for this illness in the last 12 months?

- ◯ Once
- ◯ 2-3 times
- ◯ More than 3 times
- ◯ Don't know

F9. Have the symptoms resolved? ◯ Yes ◯ No

F10. If you went to a health facility were any samples taken? IF NO, SKIP TO F12

◯ Yes ◯ No ◯ Can't remember

F11. If yes, which samples were taken?

☐ Blood ☐ Urine ☐ Stool ☐ Sputum ☐ Don't remember

F12. Were you informed of the diagnosis during that visit?

Yes ◯ No ◯ ◯ Can't remember

F13. If yes, what were you told was the diagnosis? ……………………………………………………………………………

Respiratory illness history

F14. In the last 10 days have you had an illness with cough or sore throat or running nose)?

Yes No 0Unknown

F15. Do you currently having any of the following symptoms (, cough or sore throat or running nose)?

Yes No

F16. If yes, check all that apply. IF YES TO ANY TWO OF THE SYMPTOMS, AN NP/OP SWABS WILL BE COLLECTED FROM THE PARTICIPANT

Symptom	No. of Days since onset
Fever (or history of fever)	______________________
Cough	______________________
Sore throat	______________________
Running nose	______________________

SECTION G: FOOD CONSUMPTION AND PREPARATION

G1. Do you use milk? Yes ◯ No ◯ IF NO SKIP TO G12

G2. How do you consume the milk? DO NOT PROMPT. TICK ALL THAT APPLY

☐ Tea ☐ Add to vegetables ☐ Drinking ☐ Other (specify)

G3. Where do you get the milk? TICK ALL THAT APPLY

☐ Commercial (packaged)
☐ From own animals
☐ Neighbor's animals

☐Unprocessed milk from market

G4. How often do you consume the following? USE THE FOLLOWING CATEGORIES
1-DAILY
2- WEEKLY
3-MONTHLY
4-OCCASIONALLY
5-NEVER

G5. Animal type	G6. Unboiled milk	G7. Boiled milk	G8. Fermented milk		G11.Pasteurized/packaged milk
			G9. Boiled	G10.Unboiled	
Cow					
Goat					
Sheep					
Camel					

G12.Do you consume uncooked or undercooked meat? ◯Yes ◯No

G13. If yes, how often?
◯ Daily ◯ Weekly ◯ Monthly ◯ Occasionally
◯ Other, specify_______________________________________

G14. Do you consume uncooked blood? ◯Yes ◯ No

G15. If yes, how often?
◯Daily ◯Weekly ◯Monthly ◯ Occasionally
◯ Other, specify_____________________________________

G16. Do you consume dairy products like yoghurt, cheese? ◯ Yes ◯ No

G17. If yes how often?

◯ Daily ◯ weekly ◯ monthly ◯ Occasionally

◯ Other, specify____________________________

G18. Where do you obtain the yoghurt, cheese from?

- ☐ Commercial (packaged from factory)
- ☐ Prepare at home using milk from own animals
  - ☐ Use unboiled milk
  - ☐ Use boiled milk
- ☐ Obtain from neighbor or locally produced (not pre packaged)

G19. Do you handle raw meat e.g. while preparing to cook? IF NO, GO TO H1 Yes ◯ No ◯

G20. If yes, from what animals. DO NOT PROMPT. TICK ALL THAT APPLY

- ☐ Cattle
- ☐ Goat
- ☐ Sheep
- ☐ Camel
- ☐ Wild animals

Appendix 2: Household Follow up Questionnaire

PART A. GENERAL INFORMATION

**A1. Compound head name:**			
**A2. Household head name:**			
**A3. Household ID:**		**A4.Date of interview: (dd/mm/yy)**	
**A5. Telephone of household head:**			
**A6. Enumerator initials:**			

PART II: HUMAN PARTICIPANT FORM

This will be administered to each individual enrolled

B1. Household ID:_________________
B2. Specimen ID ___________________
B3. Date of collection (dd/mm/yy) ___________________________

B4. Have you had any febrile illness in the past 3 months? IF NO OR DON'T REMEMBER SKIP TO B9

◯ Yes ◯ No ◯ Don't remember

B5. How long ago was the most recent febrile illness? ___________months (IF CURRENT FEBRILE ILLNESS INDICATE ZERO)

B6. Onset of most recent febrile illness

◯ Acute (sudden) ◯ Insidious (gradual) ◯ Cannot remember

B7. Duration of illness in days ___________

B8. What signs and symptoms did/ do you exhibit? DO NOT PROMPT. IF TICKED, INDICATE DURATION IN DAYS

- ☐ Hotness of body, Intermittent ……………………………………
- ☐ Hotness of body, Constant ……………………………………
- ☐ Chills ……………………………………
- ☐ Weight Loss ……………………………………
- ☐ Night Sweats ……………………………………
- ☐ Headache ……………………………………
- ☐ Malaise ……………………………………
- ☐ Lack of appetite ……………………………………

☐ Stiff or painful neck ……………………………….
☐ Joint or muscle pain ……………………………….
☐ Back pain ……………………………….
☐ Abdominal pain …………………………………
☐ Other(specify)

B9. Have you had a spontaneous abortion (Miscarriage) (FOR FEMALES ABOVE 15 YEARS)
◯ Yes ◯ No …………………………………

B10. Have you been treated for any febrile illness in the last 3 months? (IF NO SKIP TO B20)
◯ Yes ◯ No ◯ Unknown

B11. If yes, where were you treated?
☐ Public health facility
☐ local chemist
☐ traditional healer
☐ private clinic
☐ mobile clinic
☐ acquired medicine from
  ☐ Shop
  ☐ Friend
  ☐ Relative
  ☐ Self medication with Herbs
  ☐ Other (specify) ________________________________

B12. What medicines did you take?
☐ Antibiotics
☐ Antimalarial
☐ Pain killers
☐ Herbal
☐ Other (specify) _______________________________________
☐ Don't know

B13. For how long did you take the medication? NUMBER OF DAYS…__________………

B14. How many times have you been treated for this illness in the last 12 months?
◯ Once
◯ 2-3 times
◯ More than 3 times
◯ Don't know

B15. Have the symptoms resolved? ◯ Yes ◯ No

B16. If you went to a health facility were any samples taken? IF NO, SKIP TO F12
◯ Yes ◯ No ◯ Can't remember

B17. If yes, which samples were taken?
☐ Blood ☐ Urine ☐ Stool ☐ Sputum ☐ Don't remember

B18. Were you informed of the diagnosis during that visit?

Yes ◯ No ◯ ◯ Can't remember

B19. If yes, what were you told was the diagnosis?

………………………………………………………………………………

Respiratory illness history

B20. In the last 10 days have you had an illness with cough or sore throat or running nose)?

Yes No Unknown

B21. Do you currently having any of the following symptoms (, cough or sore throat or running nose)?

Yes No

B22. If yes, check all that apply. IF YES TO ANY TWO OF THE SYMPTOMS, AN NP/OP SWABS WILL BE COLLECTED FROM THE PARTICIPANT

Symptom	No. of Days since onset
Fever (or history of fever)	____________________
Cough	____________________
Sore throat	____________________
Running nose	____________________
