# Supplementary material for: Design of a Prospective Human–Animal Cohort Study to Evaluate the Role of Camels and Other Livestock Species in the Transmission of Brucella spp. to Humans in Kenya
Source: Int J Environ Res Public Health. 2025 Dec 12;22(12):1859. doi: 10.3390/ijerph22121859 (PMC12733042; doi:10.3390/ijerph22121859)
Supplement: Supplementary file 1 [file ijerph-22-01859-s001.zip › Supplementary Material S3_Herd Baseline_Follow-up_and Clinical Visit_Questionnaire.pdf]

Appendix 4: Enrolment Herd Questionnaire

HERD INFORMATION : *To be answered by the* ***compound head.***

A. General Information

**Date (dd/mm/yyyy):**		**Interviewer's Name:**
**Sub-location:**		**Compound ID:**
**Compound Geo-codes:**		
**Compound Head's Name (*at least two names*):**		
**Telephone:**		
**Number of households in compound:**		

B. Animal demographics

B1. Do you own any livestock (Cattle, Sheep, Goats and Camels)?

Yes *(Go to B2)* No *(Move to Baseline Household questionnaire)*

How many animals of each species do you own?

Livestock	Maturity status	B2. Number Owned	B3. Breeds	B4. Production system	B5. Usual calving season	B6. Breeding system	B7. Source of semen
**Cattle**	Calves						
	Young adults						
	Adults						
	**Total**						
**Goats**	Kids						
	Young adults						
	Adults						
	**Total**						
**Sheep**	Lambs						
	Young adults						
	Adults						
	**Total**						
**Camels**	Calves						
	Young adults						
	Adults						
	**Total**						

**Breeds:**	1 = Indigenous 2 = Exotic 3 = Cross breed
**Production system**:	1 = Settled pastoralist 2 = Agro-pastoralist 3 = Mixed farming marginal 4 = Commercial ranch 5 = Peri-urban 6 = Semi-zero grazing 7=Nomadic pastoralist
**Usual calving period:**	1 = Anytime 2 = Jan - Mar 3 = Apr – June 4 = July – Sept 5 = Oct - Dec
**Breeding system:**	1 = Artificial insemination 2 = Natural 3 = Both 4 = Don't breed
**Source of semen;**	1 = Own bull 2 = Other bull

B8 What is the main source of drinking water for your livestock?

○ Pan/pond ○ Borehole ○ River ○ Communal trough ○ Tap water
○ Other (Specify)

C. *Risk factor information*

C1 Who else owns animals in the herd other than those in this compound??

☐ Relatives ☐ Friends ☐ Neighbour ☐ No one else ☐ Other (Specify) ________________

C2 Has your herd come in contact with other herds during grazing or watering in the past 3 months?

○ Yes ○ No ○ Don't know

C3 Has your herd come in contact with wild animals during grazing or watering in the past 3 months?

○ Yes *(Go to C4)* ○ No *(Skip to C5)* ○ Don't know *(Skip to C5)*

C4 If yes, which wild animals? (*Tick all that apply*)

☐ Zebra ☐ Buffalo ☐ Antelope ☐ Waterbuck

☐ Wildebeest ☐ Other (Specify)

C5 Have you experienced any of the following signs in your livestock in the last 12 months? (*Prompt and tick all that apply*)

☐ Abortions ☐ Still births ☐ Retained placenta

☐ Swollen testes ☐ Weak calf/kid/lamb ☐ Repeat breeder

☐ Swollen joints ☐ No *(Skip to C11)*

If you have experienced abortions, stillbirths and/or weak calf/kid/lamb in the last one year, complete this table:

C6. No. of abortions	C7. No. of stillbirths	C8. No. of weak	C9. No. of animals with retained placenta	C10. Number of Live births

	calf/kid/lamb	in the last one year
**Cattle**		
**Goat**		
**Sheep**		
**Camel**		

C11 Do you own cattle?

○ Yes ○ No (*Go to C16)*

C12 Have your cows been bred by bulls belonging to another herd in the last 1 year?

○ Yes ○ No ○ I do not own cows ○ Don't know

C13 Have your bulls bred cows belonging to another herd in the last 1 year?

○ Yes ○ No ○ I do not own bulls ○ Don't know

C14 Do you use designated areas when your cows give birth?

○ Yes ○ No ○ Sometimes ○ Don't know

C15 Have you ever vaccinated your cattle herd against brucellosis?

○ Yes ○ No ○ Don't know

C16 Do you own sheep?

○ Yes ○ No (*Go to C20)*

C17 Have your female sheep been bred by male sheep belonging to another herd in the last 1 year?

○ Yes ○ No ○ I do not own female sheep ○ Don't know

C18 Have your male sheep bred female sheep belonging to another herd in the last 1 year?

○ Yes ○ No ○ I do not own male sheep ○ Don't know

C19 Do you use designated areas when your female sheep give birth?

○ Yes ○ No ○ Sometimes ○ Don't know

C20 Do you own goats?

○ Yes ○ No (*Go to C25)*

C21 Have your male goats bred female goats belonging to another herd in the last 1 year?

○ Yes ○ No ○ I do not own male goats ○ Don't know

C22 Have your female goats been bred by male goats belonging to another herd in the last 1 year?

○ Yes ○ No ○ I do not own female goats ○ Don't know

C23 Do you use designated areas when your female goats give birth?

○ Yes ○ No ○ Sometimes ○ Don't know

C24 Have you ever vaccinated your goat herd against Brucellosis?

○ Yes ○ No ○ Don't know

C25 Do you own camels?

○ Yes ○ No (*Go to C29)*

C26 Have your female camels been bred by male camels belonging to another herd in the last 1 year?

○ Yes ○ No ○ I do not own female camels ○ Don't know

C27 Have your male camels bred female camels belonging to another herd in the last 1 year?

○ Yes ○ No ○ I do not own male camels ○ Don't know

C28 Do you use designated areas when your female camels give birth?

○ Yes ○ No ○ Sometimes ○ Don't know

C29 Have you ever found aborted fetuses on the grazing pastures and watering points in the last 3 months?

○ Yes ○ No ○ Don't know

C30 How do you usually dispose aborted fetuses/still births? *(Go to Part III:D)*

○ Bury ○ I do not dispose ○ Burn

○ Throw in the bin ○ Throw in the bush ○ Don't know

○ Feed to dogs ○ Other (Specify)

Baseline Enrolled Animal Questionnaire

To be answered by ***compound head*** *and the person taking care of the animals*

Number of animals recruited: ________ animals

A. Individual Animal Details

D1 **Tag number:** ______________________________

D2 **Species:** ○ Cattle *(Skip to D3)* ○ Goat *(Skip to D4)*

		○ Sheep *(Skip to D4)*	○ Camel *(Skip to D5)*	
D3	**Age (Cattle):** *(Skip to D6)*	○ Less than 2 years	○ 2 – 3 years	○ Over 3 years
D4	**Age (Shoats):** *(Skip to D6)*	○ Less than 6 months	○ 6 – 12 months	○ Over 1 year
D5	**Age (Camels):** *(Go to D6)*	○ Less than 4 years	○ 4 – 6 years	○ Over 6 years
D6	**Breed:**	○ Indigenous	○ Exotic	○ Cross breed
D7	**Maturity status:**	○ Young	○ Young adult	○ Adult
D8	**Sex:**	○ Female *(Go to D9)*	○ Male *(Skip to D16)*	
D9	**Breeding status 1**	○ Nulliparous *(Skip to D20)*<br>○ Post-partum/lactating *(Skip to D11)*<br>○ Lactating *(Skip to D11)*	○ Pregnant *(Go to D10)*<br>○ Not pregnant *(Skip to D12)*	
D10	If pregnant, how far along is the pregnancy? *(If unknown, do pregnancy diagnosis if possible)*	______________________	Months *(Skip to D13)*	
D11	If post-partum, when was the last parturition?	______months ago *(Skip to D14)*	☐Don't know	
D11a	If lactating, is the animal milked ?	☐Yes	☐No	
D11b	If the animal is milked, how is the milk used	☐Consumed in the household	☐Sold to other households	
D12	If not pregnant, when was the last parturition? ?	______months ago *(Skip to D14)*	☐Don't know	
D13	If pregnant, how was pregnancy achieved? : *(Skip to D15)*	○ Artificial Insemination ○ Used own bull	○ Used other bull	○ Don't know
D14	If post partum or not pregnant, how was the most recent pregnancy achieved? *(Go to D16)*	○ Artificial Insemination ○ Used own bull	○ Used other bull	○ Don't know
D15	How many times has this animal been pregnant in its life time? *(Go to D16)*	○ Once ○ Twice	○ Three times	○ More than three times<br>○ Don't know
D16	**Breeding status2**	○ Young *(Skip to D20)*	○ Breeding *(Go to D17)*	○ Castrated *(Skip to D20)*
D17	If used for breeding, has this male been used for breeding within the herd in the last 1 year? *(Go to D18)* *Yes* *No* *Don't Know*			
D18	Has the male been used for breeding outside the herd in the last 1 year? *(Go to D19)* *Yes* *No* *Don't Know*			
D19	Have you ever experienced any of the following in this animal? *(Prompt and tick all that apply)* *(Go to D20)* Swollen testes Swollen joints Apparent Infertility			

D20	**Est. Weight in Kgs:**__________________		Kgs	*(Go to D21)*
D21	**Girth measurement:**	__________________	cm	*(Go to D22)*
D23	**Vaginal discharge:**	☐Clear	☐Colored (Specify)	☐Smelly
D24	**Samples collected:**	☐Serum	☐Whole blood	

Appendix 5: Routine Visit Herd Questionnaire

HERD INFORMATION : *To be answered by the* ***compound head.***

D. General Information

**Date (dd/mm/yyyy):**			**Interviewer's Name:**	
**Sub-location:**			**Compound ID:**	

E. Risk factor information

C5 Have you experienced any of the following signs in your livestock in the last 3 months? (*Prompt and tick all that apply*)

☐ Abortions ☐ Still births ☐ Retained placenta

☐ Swollen testes ☐ Weak calf/kid/lamb ☐ Repeat breeder

☐ Swollen joints ☐ No *(Skip to C11)*

If you have experienced abortions, stillbirths and/or weak calf/kid/lamb in the last one year, complete this table:

	**C6. No. of abortions**	**C7. No. of stillbirths**	**C8. No. of weak calf/kid/lamb**	**C9. No. of animals with retained placenta**	**C10. Number of Live births in the last one year**
**Cattle**					
**Goat**					
**Sheep**					
**Camel**					

Enrolled Animal

A. Individual Animal Details		
**Tag number:**	________________________________	
**Species:**	○ Cattle	○ Goat
	○ Sheep	○ Camel

Question	Response	How many days ago?
Has the animal had an abortion in the last 3 months?	☐Yes ☐No ☐Don't know	_____Days Weeks Don't know
Has the animal had a still birth in the last 3 months?	☐Yes ☐No ☐Don't know	_____Days Weeks Don't know
Has the animal delivered a weak calf in the last 3 months?	☐Yes ☐No ☐Don't know	_____Days Weeks Don't know
Has the animal had a retained placenta in the last 3 months?	☐Yes ☐No ☐Don't know	_____Days Weeks Don't know

Samples collected: ☐Whole blood

Sample ID ____________

Appendix 6: Herd Clinical Visit Questionnaire

**HERD INFORMATION :** *To be answered by the* ***compound head.***			
F. General Information			
**Date (dd/mm/yyyy):**		**Interviewer's Name:**	
**Sub-location:**		**Compound ID:**	

Clinical Case information

How was the call for the clinical case prompted?

Routine call from study staff   Case report during routine visit
Call from compound head   Other(specify)

Please indicate the species and number affected

Livestock	Cattle	Goats	Sheep	Camels
Number affected:				

	B. Individual Animal Details			
	**Is the affected animal among the tagged study animals? Yes No**			
D1	**If Yes, indicate tag number:** ____________________			
D2	**Species:**	○ Cattle	○ Goat	
		○ Sheep	○ Camel	
D3	**Age (Cattle):**	○ Less than 2 years	○ 2 – 3 years	○ Over 3 years
D4	**Age (Shoats):**	○ Less than 6 months	○ 6 – 12 months	○ Over 1 year
D5	**Age (Camels):**	○ Less than 4 years	○ 4 – 6 years	○ Over 6 years
D6	**Breed:**	○ Indigenous	○ Exotic	○ Cross breed
D7	**Maturity status:**	○ Young	○ Young adult	○ Adult

Which symptom (s) prompted the call
Abortion Still birth Delivery of weak calf Retained placenta
Others(specify) Delivery of enrolled pregnant animal

If the animal aborted, how long ago did the abortion occur? ____ days
If the animal aborted, how far along was the pregnancy? ____ months
If the animal had a still birth, how long ago did the abortion occur? ____ days
Is the placenta is not available for sampling, how was it disposed?

	How many times has this animal been pregnant in its life time?	*(Go to D16)*
	○ Once ○ Twice ○ Three times ○ Don't know	

D23	**Vaginal discharge:**	☐Clear	☐Colored (Specify)	☐Smelly
D24	**Samples collected:**	Placenta Vaginal Swab	Whole blood	
