# Supplementary material for: Design of a Prospective Human–Animal Cohort Study to Evaluate the Role of Camels and Other Livestock Species in the Transmission of Brucella spp. to Humans in Kenya
Source: Int J Environ Res Public Health. 2025 Dec 12;22(12):1859. doi: 10.3390/ijerph22121859 (PMC12733042; doi:10.3390/ijerph22121859)
Supplement: Supplementary file 1 [file ijerph-22-01859-s001.zip › Supplementary Material S4_Brucellosis Sick Visit Questionnaire.pdf]

Appendix 3: Brucellosis Sick Visit Questionnaire – Human

A. *General Information*

Participant ID **Household ID**

Date (dd/mm/yyyy): **Interviewer's Name:**

Health Facility Name:

To be answered by the ***patient.***

B. *Patient's Demographic Information*

B1. Name *(at least two names):* *(Surname, First name, Middle name)*

B2. Gender: ○ Male ○ Female

B3. Date of birth: *(dd/mm/yyyy)*

B4. Parent/Guardian name (*at least two names, if respondent under 18)* **:**

B5. Participant ID

B6. Personal/Parent's/Guardian's/Closest relative's phone number:

B7. Compound ID number:

B9 Were you referred to the facility by study staff? *(If No Go to B11)* Yes No

B10 If yes, by who?
○ During routine or clinical household visit ○ Through a call

B11 What is your employment status?(*choose one*)
○ Minor ○ Housewife ○ Student ○ Salaried-off farm skilled
○ Employed full time on farm ○ Salaried off farm non-skilled
○ Other *(specify)*

B12 What is the highest level of education you have completed? *(Choose only one)*
○ Minor ○ Currently in Primary school ○ Primary School
○ Secondary school ○ Tertiary education ○ Adult education
○ No formal education

C. *Clinical information*

C1 How many days has it been since the onset of symptoms of this current illness? ______

C2 What signs and symptoms are you currently experiencing?
(*Tick all that apply. Do not prompt. If ticked, indicate duration in days)*

Symptoms	*Duration in days*	*Symptoms*	*Duration in days*
☐ Fever, Intermittent /recurring	_________	☐ Chills	_______________
☐ Fever, constant	_________	☐ Loss of appetite	______________
☐ Headache	______________	☐ Muscle pain	______________
☐ Night Sweats	______________	☐ Fatigue	______________
☐ Abdominal pain	______________	☐ Vomiting	______________
☐ Back pain		☐ Joint pains	

C3 Does the patient have fever (37.5 ^oC or higher)? ○ Yes ○ No *(If no Go to C5)*

C4 If Yes, what is the patient's current temperature (*in ^oC*)? __________________ ºC

C5 Have you been treated for this **current illness** before ?

○ Yes *(go to C6)* ○ No (*skip to C8)*

C6 If yes in C5, where were you treated? *(Skip to C8)*

○ Another Public health facility ○ Self-medicated *(go to C7)* ○ Private clinic

○ Stand-alone chemist/pharmacy ○ Herbalist ○ Other *(specify)* __________

C7 If you self-medicated in C6 , where did you acquire the medicine from?

○ General Shop (duka/kiosk) ○ Self-medication with Herbs ○ Another person's medicine

○ Other *(specify)*

C8 Have you had an illness with symptoms similar to the current illness in the past 12 months?

○ Yes *(go to C8)* ○ No *(skip to C11)*

C9 If yes in C8, how many times have you had a similar illness over the last 12 months excluding current illness?

○ Once ○ 2-3 times ○ More than 3 times ○ Don't know

C10 Did you visit a health facility for treatment of the last illness with similar symptoms as the current illness?

○ Yes ○ No

C11 Did the symptoms of the most recent illness that was similar to this one completely resolve?

○ Yes ○ No

C12 Has any person in your household been diagnosed with brucellosis in the past one year? *(If No, skip to C14 if the patient is female, and C16 if patient is male)*

○ Yes *(Go to C13)* ○ No ○ Don't know

C13 If yes, in did you live with the person in the same household at the time they were ill?

○ Yes ○ No

C14 Have you had a miscarriage in the last 3 months? *(Ask if the female patient is above 15 years old)* Yes No

C15 If yes in C14 above, how many months was the pregnancy? ______

D. *Risk factor information- food consumption*

NOTE: These questions relate to milk consumption in the last 3 months.

D1 Do you drink milk and/or its products? Yes No (*If no, skip to section E*)

D2. Do you drink fresh goat milk?

○ Yes, less than 3 times a week ○ Yes, more than 3 times a week ○ Never (*Go to D5)*

D3. If yes in D2, how do you **usually** take fresh goat milk?

○ Boiled ○ Un-boiled ○ Both

D4. Where do you obtain fresh goat milk from?

☐ From own animals ☐ Buy packaged milk (processed)

☐ From neighbor's animals ☐ Buy from market (unpackaged)

D5. Do you consume fermented goat milk?
○ Yes, less than 3 times a week ○ Yes, more than 3 times a week ○ Never (*Go to D8)*

D6. If yes, where do you obtain the fermented milk from? (*Go to D8)*
○ Buy packaged fermented goat milk
○ Prepare at home (*Go to D7)*

D7. If you prepare fermented goat milk from your own milk at home, what do you use?
○ Use un-boiled goat milk ○ Use boiled goat milk

D8. Do you drink fresh cow milk?
○ Yes, less than 3 times a week ○ Yes, more than 3 times a week ○ Never (*Go to D11)*

D9. If yes in D8, how do you **usually** consume fresh cow milk?
○ Boiled ○ Un-boiled ○ Both

D10 Where do you obtain fresh cow milk from?
☐ From own animals ☐ Buy packaged milk (processed)
☐ From neighbor's animals ☐ Buy from market (unpackaged)

D11 Do you consume fermented cow milk?
○ Yes, less than 3 times a week ○ Yes, more than 3 times a week ○ Never (*Go to D14)*

D12 If yes, where do you obtain the fermented milk from?
○ Buy processed, packaged fermented cow milk ○ Buy unpackaged fermented cow milk
○ Prepare at home (*Go to D13)*

D13 If you prepare fermented cow milk from your own milk at home, what do you use ?
○ Use un-boiled cow milk ○ Use boiled cow milk

D14 Do you drink fresh sheep milk?
○ Yes, less than 3 times a week ○ Yes, more than 3 times a week ○ Never (*Go to D17)*

D15 If yes in D14, how do you **usually** take fresh sheep milk?
○ Boiled ○ Un-boiled ○ Both

D16 Where do you obtain fresh sheep milk from?
☐ From own animals ☐ Buy packaged milk (processed)
☐ From neighbor's animals ☐ Buy from market (unpackaged)

D17 Do you consume fermented sheep milk?
○ Yes, less than 3 times a week ○ Yes, more than 3 times a week ○ Never (*Go to D20)*

D18 If yes, where do you obtain the fermented sheep milk from?
○ Buy packaged fermented sheep milk ○ Buy unpackaged fermented sheep milk
○ Prepare at home (*Go to D19)*

D19 If you prepare fermented sheep milk from your own milk at home, what do you use?
○ Use un-boiled sheep milk ○ Use boiled sheep milk

D20 Do you drink fresh camel milk?
○ Yes, less than 3 times a week ○ Yes, more than 3 times a week ○ Never(*Go to D23)*

D21 If yes in D20, how do you **usually** take fresh camel milk?
○ Boiled ○ Un-boiled ○ Both

D22 Where do you obtain fresh camel milk from?
☐ From own animals ☐ Buy packaged milk (processed)
☐ From neighbor's animals ☐ Buy from market (unpackaged)

D23 Do you consume fermented camel milk?
○ Yes, less than 3 times a week ○ Yes, more than 3 times a week ○ Never (*Go to D26)*

D24 If yes, where do you obtain the fermented milk from?
○ Buy packaged fermented camel milk ○ Buy unpackaged fermented camel milk
○ Prepare at home (*Go to D25)*

D25 If you prepare fermented camel milk from own milk at home, what do you use?
○ Use un-boiled camel milk ○ Use boiled camel milk

D26 Do you consume Fresh milk mixed with blood (Nailanga)
○ Yes, less than 3 times a week ○ Yes, more than 3 times a week ○ Never

D27 Do you drink fresh blood?
○ Yes ○ No

E. *Risk factors information – Animal contact*

E1 Have you had any livestock in your household 3 months prior to this current illness?
○ Yes ○ No *(Skip to E5)*

E2 If yes in E1, tick which animal
☐ Cattle ☐ Sheep ☐ Goat ☐ Camel

E3 Were abortions or stillbirths cases present in your animals during the last 12 months?
○ Yes ○ No *(Skip to E5)*

E4 If yes, specify animal species and number of abortions per animal species
☐ Cattle ______________ ☐ Goat __________
☐ Sheep ______________ ☐ Camel ________

E5 Have you handled cattle on a regular basis in the past 3 months?
○ Yes ○ No *(Skip to E13)*

How often have you handled cattle in the past 3 months while….? *(Tick only one)*

E6 Feeding/watering?
○ Daily ○ Several times a week ○ Occasionally ○ Never

E7 Milking?
○ Daily ○ Several times a week ○ Occasionally ○ Never

E8 Slaughter at home?
○ Daily ○ Several times a week ○ Occasionally ○ Never

E9 Moving/herding of animals?
○ Daily ○ Several times a week ○ Occasionally ○ Never

E10 Assisting with births?
○ Daily ○ Several times a week ○ Occasionally ○ Never

E11 Removing of retained placentas?
○ Daily ○ Several times a week ○ Occasionally ○ Never

E12 Contact with aborted fetus?
○ Daily ○ Several times a week ○ Occasionally ○ Never

E13 Have you handled sheep on a regular basis in the past 3 months?

○ Yes ○ No *(Skip to E21)*

How often have you handled sheep in the past 3 months while….? *(Tick only one)*

E14 Feeding/watering?
○ Daily ○ Several times a week ○ Occasionally ○ Never

E15 Shearing?
○ Daily ○ Several times a week ○ Occasionally ○ Never

E16 Slaughter at home?
○ Daily ○ Several times a week ○ Occasionally ○ Never

E17 Moving/herding of animals?
○ Daily ○ Several times a week ○ Occasionally ○ Never

E18 Assisting with births?
○ Daily ○ Several times a week ○ Occasionally ○ Never

E19 Removing of retained placentas?
○ Daily ○ Several times a week ○ Occasionally ○ Never

E20 Contact with aborted fetus?
○ Daily ○ Several times a week ○ Occasionally ○ Never

E21 Have you handled goats on a regular basis in the past 3 months?
○ Yes ○ No *(Skip to E29)*

How often have you handled goats in the past 3 months while….? *(Tick only one)*

E22 Feeding/watering?
○ Daily ○ Several times a week ○ Occasionally ○ Never

E23 Milking?
○ Daily ○ Several times a week ○ Occasionally ○ Never

E24 Slaughter at home?
○ Daily ○ Several times a week ○ Occasionally ○ Never

E25 Moving/herding of animals?
○ Daily ○ Several times a week ○ Occasionally ○ Never

E26 Assisting with births?
○ Daily ○ Several times a week ○ Occasionally ○ Never

E27 Removing of retained placentas?
○ Daily ○ Several times a week ○ Occasionally ○ Never

E28 Contact with aborted fetus?
○ Daily ○ Several times a week ○ Occasionally ○ Never

E29 Have you handled camels on a regular basis in the past 3 months?
○ Yes ○ No *(Skip to E37)*

How often have you handled camels in the past 3 months while….? *(Tick only one)*

E30 Feeding/watering?
○ Daily ○ Several times a week ○ Occasionally ○ Never

E31 Milking?
○ Daily ○ Several times a week ○ Occasionally ○ Never

E32 Slaughter at home?
○ Daily ○ Several times a week ○ Occasionally ○ Never

E33 Moving/herding of animals?
○ Daily ○ Several times a week ○ Occasionally ○ Never

E34 Assisting with births?
○ Daily ○ Several times a week ○ Occasionally ○ Never

E35 Removing of retained placentas?
○ Daily ○ Several times a week ○ Occasionally ○ Never

E36 Contact with aborted fetus?

○ Daily ○ Several times a week ○ Occasionally ○ Never

E37 Have you been involved in slaughter and butchering wild animals in the past 3 months?
○ Yes ○ No

E38 Have you been involved in cleaning/sweeping animal barns/boma in the last 3 months? *(If No skip to E40)*
○ Yes ○ No

E39 If yes in E38, how often?
○ Daily ○ Several times a week ○ Occasionally ○ Never

E40 Have you used/handled animal manure or fresh animal waste (dung) in the past 3 months?
○ Yes ○ No *(If No Go to E42)*

E41 If Yes, how often?
○ Daily ○ Several times a week ○ Occasionally ○ Never

E42 Have you handled/worked with animal hides and skins in the past 3 months?
○ Yes ○ No (*(If No Go to Part F)*

E43 If yes, what form of hides?
○ Raw hides ○ Dry hides ○ Both

F. Laboratory Information

This information will be obtained from the study laboratory technician

F1 What samples were collected?
☐ Whole blood ☐ Serum

F2 Was a malaria test done?
○ Yes ○ No *(Skip to F4)*

F3 If yes in F2 what was the result of the malaria test?
○ Positive ○ Negative

F4 Did you carry out a facility brucellosis test?
○ Yes ○ No *(Skip to F6)*

F5 If yes in F4 what was the result of facility brucellosis test?
○ Positive ○ Negative

F6 What was the result of the Rose Bengal Test?
○ Positive ○ Negative
