# Supplementary material for: Design of a Prospective Human–Animal Cohort Study to Evaluate the Role of Camels and Other Livestock Species in the Transmission of Brucella spp. to Humans in Kenya
Source: Int J Environ Res Public Health. 2025 Dec 12;22(12):1859. doi: 10.3390/ijerph22121859 (PMC12733042; doi:10.3390/ijerph22121859)
Supplement: Supplementary file 1 [file ijerph-22-01859-s001.zip › Supplementary Material S5_Human-Animal Contact Questionnaire.pdf]

Appendix 7: Human Animal Contact Questionnaire

Date: __________________

Participant ID: __________________

Have you milked, fed, slaughtered or herded any livestock animals in the last 2 weeks?
☐ Yes ☐ No

IF YES, COMPLETE THE TABLE BELOW

**Livestock**	**Type of contact**	**Frequency of contact**	**Place of contact**
Camel	☐Feeding/watering<br>☐Milking<br>☐Slaughter/handle meat<br>☐Herding<br>☐Assisting with birthing<br>☐Removal of retained placentas<br>☐Contact with aborted fetu	☐5-7 days a week<br>☐3-4 days a week<br>☐1-2 days a week<br>☐Less than once a week	☐Home<br>☐Fora<br>☒Neighbor
Cattle	☐Feeding/watering<br>☐Milking<br>☐Slaughter/handle meat<br>☐Herding<br>☐Assisting with birthing<br>☐Removal of retained placentas<br>☐Contact with aborted fetu	☐5-7 days a week<br>☐3-4 days a week<br>☐1-2 days a week<br>☐Less than once a week	☐Home<br>☐Fora<br>☐Neighbor
Sheep	☐Feeding/watering<br>☐Milking<br>☐Slaughter/handle meat<br>☐Herding<br>☐Assisting with birthing	☐5-7 days a week<br>☐3-4 days a week<br>☐1-2 days a week<br>☐Less than once a week	☐Home<br>☐Fora<br>☐Neighbor

	☐Removal of retained placentas<br>☐Contact with aborted fetu		
Goat	☐Feeding/watering<br>☐Milking<br>☐Slaughter/handle meat<br>☐Herding<br>☐Assisting with birthing<br>☐Removal of retained placentas<br>☐Contact with aborted fetu	☐5-7 days a week<br>☐3-4 days a week<br>☐1-2 days a week<br>☐Less than once a week	☐Home<br>☐Fora<br>☐Neighbor
