# Supplementary material for: Design of a Prospective Human–Animal Cohort Study to Evaluate the Role of Camels and Other Livestock Species in the Transmission of Brucella spp. to Humans in Kenya
Source: Int J Environ Res Public Health. 2025 Dec 12;22(12):1859. doi: 10.3390/ijerph22121859 (PMC12733042; doi:10.3390/ijerph22121859)
Supplement: Supplementary file 1 [file ijerph-22-01859-s001.zip › Supplementary Table S1_Study Site Characteristics.pdf]

**Supplementary Table S1: Study Site Characteristics**

| Characteristic | Marsabit County | Kajiado County | Reference |
|---|---|---|---|
| Climate classification | Arid | Semi-arid | Kenya Meteorological Department |
| Mean annual rainfall | 200-400mm | 400-600mm | [24] |
| Primary livestock species | Cattle, camels, goats, sheep | Cattle, goats, sheep | [24] |
| Camel-rearing households | >75% | 0% | [24] |
| Population density | 12 people/km² | 35 people/km² | Kenya National Bureau of Statistics |
| Pastoralism dependence | >80% households | >75% households | [24] |
